# Attenuation of acute kidney injury in a murine model of neonatal *Escherichia coli* sepsis

**DOI:** 10.3389/fcimb.2024.1507914

**Published:** 2025-02-03

**Authors:** Esther M. Speer, Atilade A. Adedeji, Joyce Lin, Alexandra Khorasanchi, Asma Rasheed, Maya Bhat, Kelly Mackenzie, Randolph Hennigar, Kimberly J. Reidy, Robert P. Woroniecki

**Affiliations:** ^1^ Department of Pediatrics, Renaissance School of Medicine at Stony Brook University, Stony Brook, NY, United States; ^2^ Department of Chemistry, Stony Brook University, Stony Brook, NY, United States; ^3^ Department of Pathology, Renaissance School of Medicine at Stony Brook University, Stony Brook, NY, United States; ^4^ Division of Pediatric Nephrology, Department of Pediatrics, Children’s Hospital at Montefiore, Bronx, NY, United States

**Keywords:** neonatal sepsis, acute kidney injury (AKI), inflammatory response, kidney injury markers, anti-inflammatory agents, pentoxifylline, *Escherichia coli sepsis*

## Abstract

**Introduction:**

Sepsis is a risk factor for acute kidney injury (AKI) in neonates, for which no effective treatment exists. The phosphodiesterase inhibitor pentoxifylline (PTX) has demonstrated renal protection from ischemia and inflammation in adult rodents. We hypothesized that addition of PTX to antibiotics may attenuate immune and histological AKI in a murine neonatal sepsis model.

**Methods:**

Postnatal (PN) day 1 C57BL/6J mice were injected with *E. coli* K1 strain at 10^5^ colony forming units per gram weight or saline control. After 1.5 hours, septic pups randomly received saline, gentamicin or cefotaxime, with/without PTX. 5.5h after sepsis initiation, kidneys and blood were harvested for measurements of biomarkers of inflammation and kidney injury. Renal sections from PN7 mice were used for histology and immunofluorescence. Linear mixed effect models were employed to fit the outcomes including interaction between treatment group and sex.

**Results:**

Septic mice demonstrated robust expression of pro-inflammatory cytokines, chemokines and biomarkers of tubular injury in renal tissue, which were attenuated in response to combined PTX and antibiotics (gentamicin or cefotaxime): chemokines (p<0.001), plasma (p<0.01) and tissue IL-6 (p<0.05), plasma TNF (p<0.001), NGAL (p<0.01), CXCL10 (p<0.01), osteopontin (p<0.05), and VEGF (p<0.05), with a trend for KIM-1 (tissue concentration: p=0.21, fluorescence area: p=0.12). Interactions between treatment and sex were present for several cytokines and kidney injury biomarkers. Immunofluorescence findings for the tubular injury markers (NGAL and KIM-1) were consistent with biomarker expression in tissue lysates.

**Conclusion:**

Neonatal *E. coli* sepsis leads to increased expression of renal tissue inflammation and injury biomarkers consistent with AKI, which may be attenuated with PTX combined with antibiotic treatment.

## 1 Introduction

Sepsis in newborns is a common and devasting condition. ~700,000 newborns die annually worldwide during their first four weeks of life due to severe infections including sepsis, the risk of which is particularly high among preterm newborns who represent over 10% of all live births globally (Lawn et al., 2005; Stoll et al., 2015; Ohuma et al., 2023; Perez et al., 2023). The distinct neonatal inflammatory immune response to sepsis, while key to reducing microbial invasion, has been associated with multi-organ dysfunction including acute kidney injury (AKI). Neonatal sepsis is one of the most common risk factors for AKI among preterm and term newborns, contributing up to 78% of the cases of AKI, further increasing its mortality and potentially impacting on long-term renal function among survivors (Momtaz et al., 2014; El-Badawy et al., 2015; Daga et al., 2017; Charlton et al., 2019; Coggins et al., 2021; Hingorani et al., 2021; Hu et al., 2021; Gohiya et al., 2022). Neonatal sepsis triggers an intense host inflammatory response, hypoperfusion and/or shock, oxidative stress and impaired mitochondrial function (Blatt et al., 2014; Poggi and Dani, 2018), all of which may contribute to increased mortality and morbidity including AKI. Currently available antimicrobial therapies alone do not prevent AKI (Selewski et al., 2015), and may even exert direct nephrotoxic effects (Salerno et al., 2021), and no alternative proven effective preventive or therapeutic pharmacological agents are available to date.

The neonatal immune system and the kidneys undergo substantial developmental and adaptive changes during the perinatal period in both term and especially in preterm newborns, creating unique challenges and susceptibilities to sepsis-induced hyperinflammation, infection-induced organ injury, and drug-induced nephrotoxicity that cannot be extrapolated from older age groups to the neonatal period (Adkins et al., 2004; Henneke and Berner, 2006; Stelloh et al., 2012; Hofer et al., 2013; Saint-Faust et al., 2014; Seely, 2017; Carpenter et al., 2023). Nephrogenesis occurs until 34 to 36 weeks gestation in humans, with 60% of nephrons forming during the third trimester (Carpenter et al., 2023), at a time when many preterm neonates encounter loss of homeostasis and instability of their organ systems, further aggravated by acute neonatal sepsis and sepsis-induced hyperinflammation. Septic preterm human newborns or neonatal mice during their first 3 days of life are at risk of low nephron endowment, nephron loss, and/or maturational delay of nephron development (Stelloh et al., 2012; Saint-Faust et al., 2014). Low nephron numbers combined with neonatal sepsis and its accompanying systemic inflammatory response syndrome, loss of homeostasis, and metabolic energy impairment during a time of rapid renal and immunological adaptation to extrauterine life can lead to substantial acute and potentially lasting kidney injury. Preclinical models to investigate the developmentally-determined factors of susceptibility to neonatal sepsis-induced AKI and to develop treatment strategies adapted to the characteristics of early neonatal life are thus needed in order to overcome these challenges.

The candidate adjunctive sepsis therapeutic pentoxifylline (PTX) (Pammi and Haque, 2023), a methylxanthine derivative and tumor necrosis factor inhibitor, may be a potential promising agent to mitigate neonatal sepsis-induced AKI. Besides inhibiting pro-inflammatory cytokine and chemokine production while enhancing the expression of endogenous anti-inflammatory interleukin (IL)-10 in newborn blood plasma (Speer et al., 2017), it has been shown to promote mitochondrial biogenesis and integrity, and demonstrated antioxidant capability in the brain (Khedr et al., 2019; Wang et al., 2020; Wang et al., 2021). In adult rodent studies, PTX has demonstrated renal protection from ischemia and endotoxinemia (Wang et al., 2006; Bansal et al., 2009; Wystrychowski et al., 2014), while other human and rodent studies have provided evidence for its protective effects against drug-induced nephrotoxicity (Stojiljkovic et al., 2009; Nasiri-Toosi et al., 2013).

We employed a murine neonatal sepsis model with intravenous (iv) or intraperitoneal (ip) injection of live *Escherichia coli* K1 strain into pups less than 24 hours old (Speer et al., 2020), to study sepsis-induced AKI and its response to antimicrobial and adjunctive anti-inflammatory treatment. Both routes of bacterial administration represent important types of neonatal sepsis, thus increasing the applicability of our study findings. Assessment of neonatal AKI in pre-clinical and clinical studies has proven to be challenging, owing to the low sensitivity of serum or plasma creatinine concentrations in human newborns as well as neonatal rodents. Novel biomarkers in serum, plasma and urine, some of which can be detected earlier, longer and with increased sensitivity compared to serum creatinine, are now available, such as serum, plasma, or urinary neutrophil gelatinase-associated lipocalin (NGAL, also known as lipocalin-2), cystatin C, or kidney injury molecule-1 (KIM-1) (Askenazi, 2012; Otto et al., 2013; Askenazi et al., 2016; Sweetman, 2017). Whereas serum creatinine concentrations depend on muscle mass in addition to renal excretion, Hari et al. (Hari et al., 2007). demonstrated that serum cystatin C was comparable between normally nourished and malnourished children, thus making it potentially more suitable and sensitive for AKI assessment of septic human or animal newborns affected by diminished growth. We therefore measured concentrations of a panel of cytokines and chemokines as well as kidney injury biomarkers in renal tissue and plasma to assess the sepsis-induced inflammatory response and acute kidney injury in our preclinical neonatal sepsis-induced AKI model.

Based on these reports, we employed this model of neonatal sepsis-induced AKI to study the hypothesis that PTX, when administered in combination with antibiotics to *E. coli-*septic neonatal mice, may inhibit expression of renal tissue biomarkers of kidney injury and inflammation and diminish histological signs of kidney injury.

## 2 Materials and methods

### 2.1 Preparation of microorganisms

Live *E. coli* K1 strain [(# 700973, American Type Culture Collection; Manassas, VA) or a bioluminescent K1 strain A192PP-lux2, which was originally derived from the neonatal septicemia clinical isolate E. coli A192 with subsequent introduction of the lux operon, as described by Witcomb et al (Witcomb et al., 2015)] were used to create our murine neonatal sepsis model (Speer et al., 2020). In brief, single colonies, stored at 4◦C on Luria-Bertani **(LB)** agar plates, were grown overnight in LB media in a shaker (150 RPM, 37◦C) to stationary phase, diluted 1:100, and grown for an additional 2 hours to exponential phase. Colony forming units **(CFU)** per ml were determined spectrophotometrically at 600 nm and confirmed by plating of serial dilutions and manual counting, as previously described by us (Speer et al., 2020). Bacterial suspensions were diluted in sterile saline to the desired inoculum concentration.

### 2.2 Neonatal mouse sepsis model

The animal protocol was reviewed and approved by the Institutional Animal Care and Use Committee, Stony Brook University, Stony Brook, NY. C57BL/6J female and male breeders from *Jackson Laboratories (Bar Harbor, ME)* were mated in-house, and pups delivered naturally at term. Animals were fed ad libitum with a standard chow diet, and maintained in a year-around climate-controlled environment with a 12-hour light-dark cycle. Pups of both sexes were used for all experiments, and remained with their dams except for brief interruptions due to experimental procedures. BSL 2 containment was practiced for all experiments involving live bacteria.

**Figure 1:**
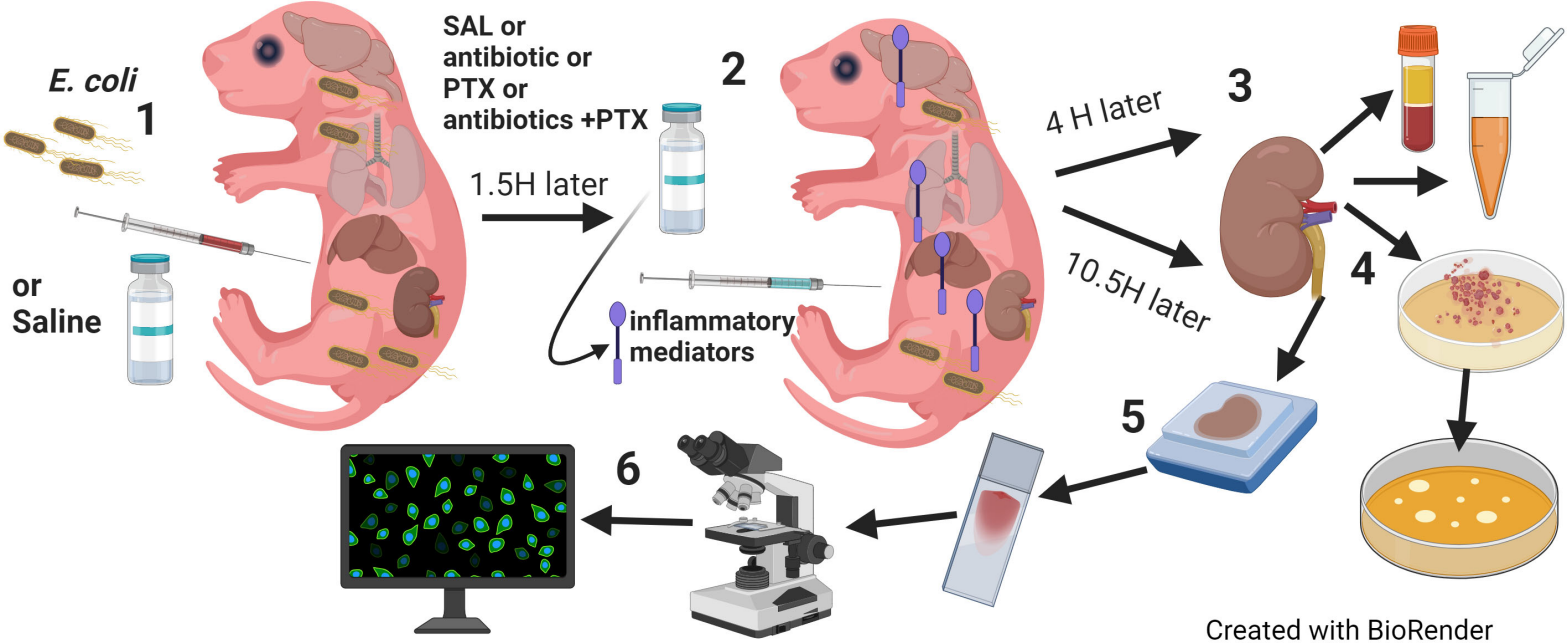
Model of neonatal sepsis-induced AKI. (1) Newborn mice less than 24 hours old were injected intravenously or intraperitoneally with *E. coli* 105 CFU/g weight or an equal volume of sterile saline for controls. (2) 1.5 hours later, pups were randomly assigned to injection with antibiotic (GENT or CEF), PTX, (antibiotic + PTX) or saline. (3) After additional 4 hours, blood and renal tissue were obtained for (4) bacterial plating and measurements of inflammatory and kidney injury biomarkers. (5) 12 hours after bacterial injections, whole kidneys were harvested and slides were prepared after formalin fixation and paraffin embedding and stained for routine (H&E, PAS) or (6) immunofluorescence microscopy, and then quantitatively analyzed for fluorescence intensity and area of fluorescence.

Newborn pups less than 24 hours old, immune and renal developmentally comparable to preterm human neonates (Adkins et al., 2004; Carpenter et al., 2023), were injected iv [via the external jugular vein (Speer et al., 2020)] or ip with live *E. coli* A192PP-lux2 or *E. coli* K1 strain (ATCC 700973), respectively, at 10^5^ CFU per g body weight or an equal volume of saline control. Pups were then treated ip with either gentamicin (GENT) (5 μg per g body weight; *Fresenius Kabi; Lake Zurich, IL*) or the non-nephrotoxic antibiotic cefotaxime **(CEF)** (25 μg per g body weight; *GoldBio; St. Louis, MO*), PTX (60 μg per g body weight; *VWR International, LLC; Buffalo Grove, IL*), combined antibiotic and PTX, or an equal volume of sterile saline control, starting at 1.5 hours after sepsis initiation. All pharmacological agents were United States Pharmacopeia (USP) grade, and 32G Hamilton syringes (*Hamilton Company; Reno, NV*) were used to enable dosing at 1 μl precision for all injections of bacteria and pharmacological agents. Upon completion of their respective observation period, as detailed for each experiment, pups were euthanized, and blood and renal tissues harvested under sterile conditions to assess for the presence of sepsis-induced inflammation and acute kidney injury changes as well as bacterial growth (see **Figure 1** for a schematic representation of our treatment protocol). Sex of newborn pups was determined postmortem through genotyping of mouse tissue for the presence of the Y chromosome (*Transnetyx; Cordova, TN*).

The adequacy of iv injections into less than 24 hours-old pups was confirmed qualitatively through mixing of low concentration (0.05%) Evans blue dye to the bacterial inoculum prior to injections, as previously described by us (Speer et al., 2020), whereby successful iv bacterial injections led to homogenous discoloration of neonatal mice as opposed to local discoloration at the injection site due to extravasation (Speer et al., 2020). Only high-quality iv injections were included for further analyses. In addition, we quantitatively measured photon emission of the bioluminescent *E. coli* strain A192PP-lux2 soon after iv bacterial injections within regions of interest around the injection site using an IVIS Lumina III *In Vivo* Imaging System (*PerkinElmer; Waltham. MA*) [see (Speer et al., 2020) for a more detailed description of the methodology and its validation]. Brief sedation/anesthesia with isoflurane was used during *in vivo* imaging and as indicated for injections. Images of all injected animals were obtained for documentation of color changes and bioluminescence.

### 2.3 Initial processing of blood and renal tissue and bacterial culturing

Blood was immediately mixed with sodium citrate and kept on ice. An aliquot of each blood sample was used for bacterial plating of serial dilutions on LB agar plates, whereas the remaining blood was centrifuged (500 g, 10 min, room temperature), and plasma was removed and stored at −80°C until further assayed.

Kidneys were immediately placed into 1.7 ml microcentrifuge tubes pre-filled with 500 μl sterile endotoxin-free saline and kept on ice. Organ weight was determined by subtracting the measured tube weight prior to and after addition of tissue with a Mettler Toledo AT261 Delta Range Analytical Balance (*Mettler Toledo; Columbus, OH*). Sterile 2.3 mm zirconia/silica beads (*Biospec Products Inc.; Bartlesville, OK*) were then added to renal tissue for homogenization with a BeadBug Mini Homogenizer (*Benchmark Scientific, Inc.; Sayreville, NJ*), as previously described (Speer et al., 2020). An aliquot of each renal tissue homogenate was subjected to bacterial plating, and the remaining tissue samples were centrifuged (13,000 g, 10min, 4 °C), and supernatants harvested and stored at −80°C.

Agar plates containing blood and tissue homogenates were incubated at 37°C in a humidified incubator for ∼18–24 hours, and microbial colonies were determined through manual counting with an eCount Colony Counter (*Heathrow Scientific; Vernon Hills, IL*) and expressed as CFUs per ml blood or CFUs per mg organ tissue, respectively, as previously described (Speer et al., 2020).

### 2.4 Measurement of cytokine and chemokine concentrations in plasma and supernatants of tissue homogenates

A panel of pro- and anti-inflammatory cytokines and chemokines was tested in blood plasma and tissue homogenate supernatants with Bio-Plex Pro Multiplex Immunoassays (*Bio-Rad Laboratories; Hercules, CA*) and analyzed on a Bio-Plex 200 system with Bio-Plex Manager 5.0 software (*Bio-Rad*). Results were expressed as cytokine concentrations in pg per ml plasma and pg per mg protein for renal tissue homogenate supernatants, respectively. Duplicate technical replicates were used for all immunological studies. Protein concentrations in tissue homogenate supernatants were determined with the Bradford method (*Bio-Rad*) and assayed on a Spectramax 190 Plate Reader (*Molecular Devices LLC; San Jose, CA*).

### 2.5 Analysis of kidney injury biomarkers in tissue homogenate supernatants

In order to examine the expression of biomarkers for sepsis-induced kidney injury, we measured the concentrations of analytes of the MILLIPLEX MAP Mouse Kidney Injury Magnetic Bead Panels 1 and 2 (*MilliporeSigma; Burlington, MA*), which included NGAL, KIM-1, and cystatin C, in renal tissue lysate supernatants (Sweetman, 2017), as per manufacturer’s recommendation, using the Bio-Plex 200 system.

### 2.6 Histology of renal tissue

In order to examine renal tissue histologically for any acute structural changes, we harvested kidneys from 7 days old *E. coli*-septic pups 12 hours after sepsis-initiation that were treated or not with antibiotics (CEF) or combined (CEF and PTX) *vs* healthy controls. We chose this later time point as opposed to the early collection of samples for acute inflammatory and renal injury marker measurements, as 12 hours of sepsis was the duration until which the majority of septic pups were able to survive, while still allowing some time for the potential occurrence of acute tissue changes. Likewise, 7 days old mice were used for histological experiments, since these mice are more robust with an expected longer survival duration compared to 1-day old pups. In addition, the kidney size of 7 days old mice makes preparation of multiple histology and immunohistochemistry stains from the same kidneys more feasible. Formalin-fixed and paraffin-embedded 5µm thick sections were stained with hematoxylin and eosin (H&E) and periodic acid Schiff (PAS). These tissue stains were microscopically evaluated by a renal pathologist blinded to the treatment status of the animals, noting pattern, location and degree of any noticeable acute kidney injury changes. 3 septic non-survivors with markedly changed postmortem images (H&E and PAS) were excluded from further analyses.

### 2.7 Immunofluorescence staining for kidney injury biomarkers and quantitative image analysis

We further employed immunofluorescence techniques to stain for specific markers of tubular injury, and evaluated these tissue sections both qualitatively and quantitatively. Slides with mounted formalin-fixed and paraffin-embedded (FFPE) tissue sections were baked for 1 hour at 65°C. Slides were then deparaffinized with Xylene, followed by washes with decreasing concentrations of ethanol (100%, 95%, 70%) and deionized water for rehydration. Following this step, slides were immersed in citrate buffer solution and placed in a pressure cooker at 120°C for 10 minutes. After cooling, slides were washed in Tris buffered saline with tween 20 (TBST), followed by blocking in 2% non-fat milk for 1 hour. Slides were then incubated with primary antibodies overnight at 4°C. These comprised of rabbit recombinant monoclonal anti-mouse lipocalin-2/NGAL/clone EPR21092 (cat. number: 216462; *Abcam; Waltham, MA*) at 1:200 dilution or antigen affinity-purified polyclonal anti-mouse KIM-1 antibody (cat. number: AF1817; R&D Systems; Minneapolis, MN) at 1:200 dilution. The next day, slides were washed three times in TBST, incubated with goat anti-rabbit IgG secondary antibody (cat. number: 111-005-144; *Jackson Immuno Research Laboratories, Inc.; West Grove, PA*) at 1:300 dilution for 30 minutes at 37°C, washed again three times with TBST, followed by incubation with Alexa Fluor 568-conjugated donkey anti-goat IgG antibody (cat. number: A11057; *Invitrogen Corp.; Waltham, MA*) at 1:300 dilution for 30 minutes at 37°C. After three washes with TBST, slides were incubated with Hoechst (cat. number: 83219; *Anaspec, Inc.; Fremont, CA*) at a dilution of 1:1,000 for 10 minutes at room temperature, washed three times with TBST, and incubated with fluorescein-conjugated *Lotus Tetragonolobus* lectin (*Vector Laboratories, Inc.; Newark, CA*) staining at 1:100 dilution at room temperature for 10 minutes. This was succeeded by two final washes with TBST, before mounting the coverslip. Whole microscopic images were scanned on a Nikon Eclipse 90i microscope at 20x magnification, using the same settings for all slides for the respective analyte of interest. Subsequently, scanned images were analyzed with ImageJ (https://imagej.nih.gov). After drawing a region of interest that carefully outlined the renal cortex, the mean fluorescence intensity as well as the percentage of the area stained above threshold were determined for each kidney section. Both mean fluorescence intensity and percentage of stained area for each kidney injury marker and each individual kidney section were then compared between the different treatment groups.

### 2.8 Statistical analysis

All microbial counts were expressed as CFUs per ml vs. CFUs per mg tissue for plasma and organ tissue samples, respectively, and all biomarkers of kidney injury and inflammation including cytokine and chemokine concentrations were expressed as pg per ml plasma *vs.* pg per mg protein for supernatant tissue homogenates. Means and standard errors were estimated for normally distributed data, and median and interquartile ranges (IQR) for non-normally distributed data, whereby the assumption of normality was assessed graphically using Q-Q-plots and through the application of Kolmogorov-Smirnov tests for normal distribution. Group comparisons employed one-way ANOVA or Kruskal-Wallis tests for multiple group comparisons corrected by false discovery rates for parametric and non-parametric data, respectively. Unpaired Welch t-tests, which do not assume variances homogeneity, and Mann Whitney U-tests were used for pair-wise group comparisons, as indicated.

Linear mixed effect models considering the litter as a random effect were used to fit the outcomes. The fixed effects of the models included the treatment group (GENT *vs* PTX *vs* GENT and PTX *vs* SAL) and sex (female *vs* male). A second set of linear mixed effect models were further constructed with adjustment of an interaction term between treatment group and sex in order to model the possible gender effect on the difference of outcome values between treatment groups. For both sets of models, the log-transformation was applied on certain outcome values to meet the normality assumption based on the residual distribution. Analyses were performed using SAS 9.4 (*SAS Institute Inc., Cary, NC*) as well as GraphPad Prism Version 9.2.0 (*GraphPad Software; San Diego, CA*) for analyses and for graphing of results. All statistical tests were two-sided and p < 0.05 were determined significant.

## 3 Results

### 3.1 Treatment of *E. coli* septic newborn mice with PTX does not enhance bacterial growth

**Figure 2:**
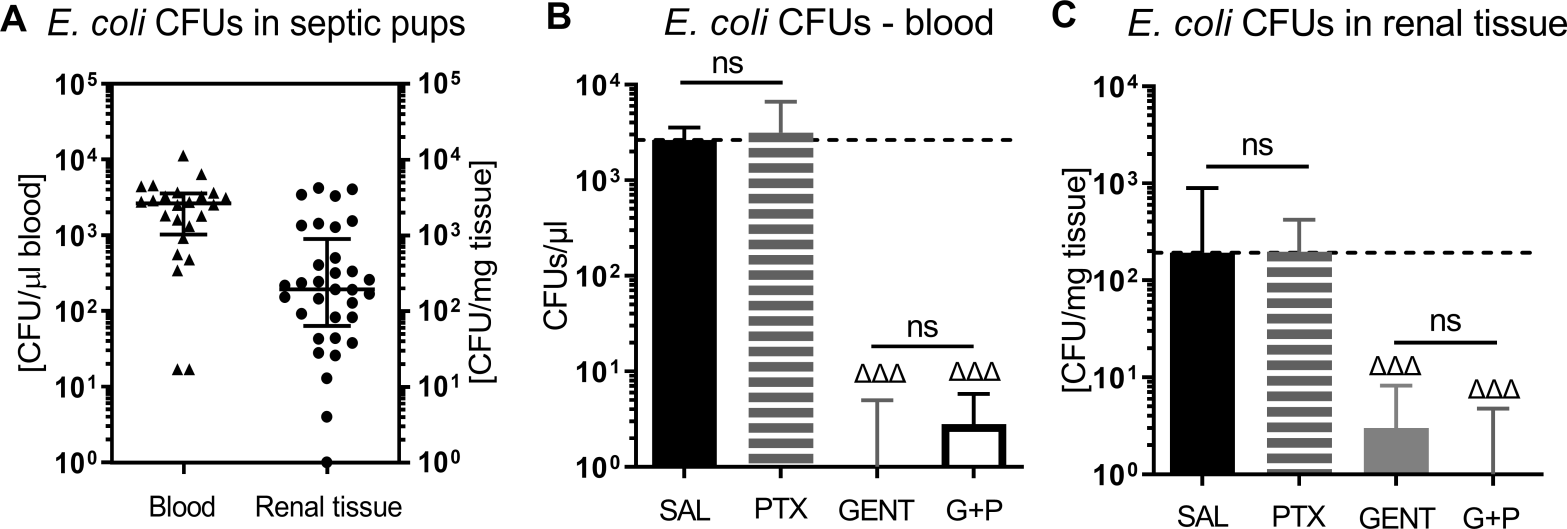
*E. coli* bacterial growth in blood and renal tissue of septic newborn mice. Live *E. coli* at 10^5^ CFU per g body weight were injected iv into less than 24 hours old pups, and bacterial CFUs were determined from blood and renal tissue after 5.5 hours of sepsis initiation. Panel **(A)** shows bacterial growth in blood and renal tissue of untreated septic mice, demonstrating that bacteria were adequately distributed in our newborn sepsis mouse model. **(B)** shows CFUs in blood from untreated septic mice (SAL, n = 24) and pups treated with PTX (n = 20), GENT (n = 17), or (GENT and PTX [G+P], n = 20). **(C)** demonstrates CFUs in renal tissue from untreated septic mice (n = 33), and mice treated with PTX (n = 23), GENT (n = 18), and G+P (n = 24). Median CFUs in septic untreated mice were represented through the interrupted line on panels **(B, C)**. Significant concentration differences between treated *vs* untreated septic mice were indicated through triangles above the respective treatment column, ΔΔΔ p<0.001, non-significant: ns.

As can be seen in **Figure 2A**, *E. coli* CFUs can be retrieved from cultures of blood and renal tissue homogenates from neonatal mouse pups less than 24 hours old that were injected iv with 10^5^ CFUs of live *E. coli*, whereby a high density of CFUs was present in the blood of septic animals. **Figures 2B, C** demonstrate the treatment effects on bacterial growth. As expected, GENT suppressed bacterial growth in blood and renal tissue, whereas addition of PTX to untreated septic or GENT-treated mice did not alter their bacterial growth. Comparable findings were achieved after ip injection of *E. coli* followed by treatment with CEF and/or PTX (data not shown).

### 3.2 *E. coli* sepsis in neonatal mice induced a distinct and robust systemic and renal tissue inflammatory cytokine response that was modified in response to GENT and/or PTX

**Figure 3:**
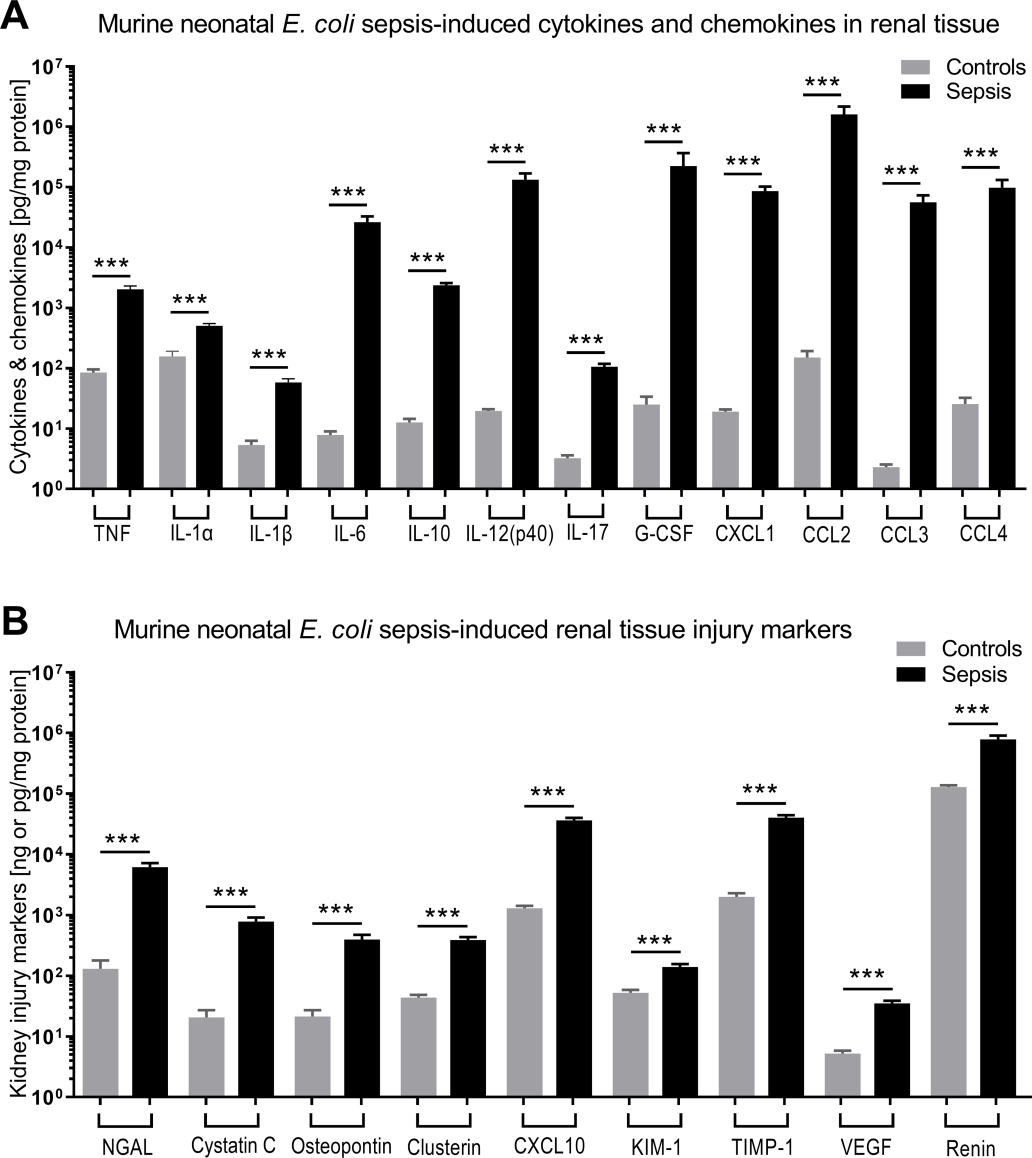
*E. coli* sepsis led to robust cytokine and chemokine responses and expression of kidney injury markers in renal tissue of newborn mice. Neonatal mice pups less than 24 hours old were injected iv with live 10^5^ CFU *E. coli* per g body weight (sepsis, n=33) or an equal volume of sterile saline (controls, n=26), and subsequently left untreated for 5.5 hours. **(A)** Cytokine and chemokine concentrations measured in renal tissue lysate supernatants and **(B)** kidney injury biomarkers are shown. Significant concentration differences between uninfected controls and untreated septic mice were indicated through stars above connecting lines. NGAL, cystatin C, osteopontin, and clusterin in panel **(B)** are displayed in ng/mg protein, whereas the other kidney injury markers in panel **(B)** are shown in pg/mg protein. Chemokine (C-X-C motif) ligand 1 (CXCL-1), chemokine (C-C motif) ligand 2 (CCL2), ***p<0.001.

**Figure 4:**
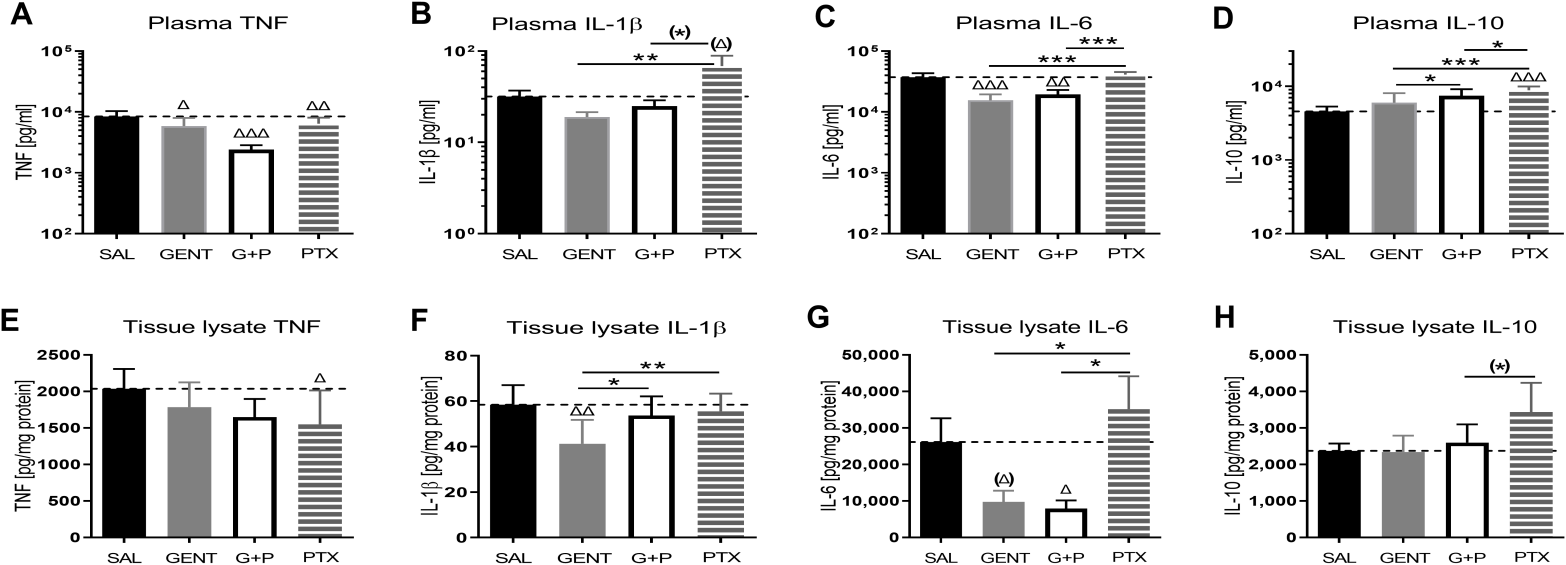
*E. coli* sepsis induced a distinct systemic and renal tissue pro-inflammatory cytokine response in neonatal mice that was mitigated with GENT and/or PTX. Injection of live *E. coli* (10^5^ CFU per g body weight iv) to newborn mice less than 24 hours old induced high concentrations of pro- and anti-inflammatory cytokines, notable TNF **(A, E)**, IL-1β **(B, F)**, IL-6 **(C, G)**, and IL-10 **(D, H)**, in blood plasma [panels **(A–D)**] and homogenized renal tissue [panels **(E–H)**], that showed responses to treatment of mice with GENT (n = 18), PTX (n = 23), or (GENT and PTX [G+P], n = 24) compared to untreated septic mice (SAL, n = 33). Mean measured cytokine concentrations in septic untreated mice were represented through the interrupted line on each panel. Significant concentration differences between treated vs untreated septic mice were indicated through triangles above the respective treatment column, whereas significant differences between treatment options were indicated through stars above connecting lines. (*) noticeable but non-significant difference p≥0.05, *p<0.05, **p<0.01, ***p<0.001, (Δ) noticeable but non-significant difference p≥0.05, Δ p<0.05, ΔΔ p<0.01, ΔΔΔ p<0.001. **(B)**: p= 0.069 for (Δ) and p=0.062 for (*); **(G)**: p=0.102 for (Δ); and **(H)**: p=0.053 for (*).

There were 124 mice from 36 litters in this dataset, with an average weight of 1.5g, whereby 33 mice belonged to the sepsis group, 18 mice were exposed to GENT alone, 23 mice received PTX alone, 24 mice received combined (GENT and PTX) treatment, and 26 mice served as uninfected controls. Compared to non-septic controls, *E. coli* septic-pups demonstrated markedly increased expression of all cytokines, chemokines, and kidney injury biomarkers measured (p < 0.001) (**Figure 3**). These findings provide evidence for sepsis initiation and its accompanying inflammatory response syndrome in our model. Only one male pup treated with combined GENT and PTX died prior to completion of this short-term observation period. *E. coli* sepsis led to rapid and robust increased expression of pro- and anti-inflammatory cytokines in blood plasma and renal tissue [**Figure 4** *;* see also (Speer et al., 2020) for cytokine responses to murine neonatal sepsis in other peripheral organs]. Whereas GENT, PTX, and combined (GENT and PTX) inhibited TNF expression in plasma, PTX and (GENT and PTX) enhanced plasma IL-10 concentrations. Comparable changes were however not observed in renal tissue of *E. coli*-septic mice. This may in part be due to sex differences in cytokine responses to these treatments (see below) as well as differences in the timing of systemic *vs.* peripheral organ inflammatory responses. The pro-inflammatory as well as pro-resolution cytokine IL-6 led to a comparable pattern in plasma and renal tissue lysates. This was characterized by diminished GENT- and (GENT and PTX)-induced IL-6 concentrations in plasma and in response to (GENT and PTX) in renal tissue with a non-significant trend towards lower IL-6 with GENT treatment. By contrast, only GENT significantly suppressed the inflammasome-associated cytokine IL-1β compared to untreated sepsis, PTX or (GENT and PTX) in renal tissue and compared to PTX alone in blood plasma.

Furthermore, IL-12(p40), IL-17, and G-CSF concentrations in renal tissue lysates of *E. coli* septic newborn mice were all significantly suppressed when treated with GENT or (GENT and PTX) compared to untreated septic mice, whereas IL-1α showed a non-significant trend towards lower renal tissue concentrations with (GENT and PTX) (see **Supplementary Figure S1**).

### 3.3 Combined GENT and PTX profoundly inhibited renal tissue chemokine expression in murine neonatal *E. coli* sepsis

**Figure 5:**
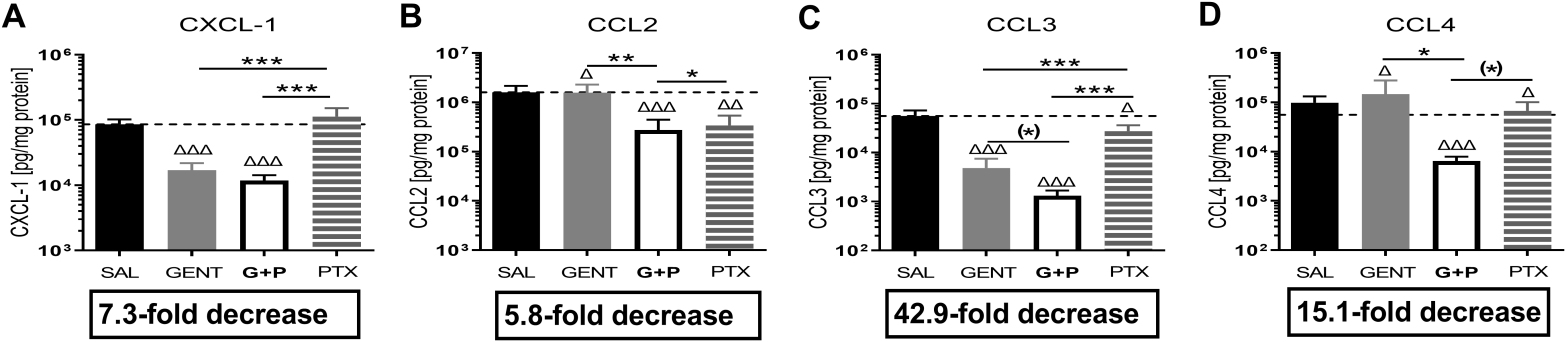
Combined GENT and PTX profoundly inhibited renal tissue chemokine expression in murine neonatal *E. coli* sepsis. Neonatal mice pups less than 24 hours old were injected iv with live 10^5^ CFU *E. coli* per g body weight, and treated with GENT (n = 18), PTX (n = 23), combined (GENT and PTX [G+P], n = 24), or left untreated (SAL, n = 33) 1.5 hours after bacterial injections and observed for further 4 hours. Chemokine concentrations measured in renal tissue lysate supernatants for **(A)** CXCL-1, **(B)** CCL2, **(C)** CCL3, and **(D)** CCL4 are shown. Mean chemokine concentrations in septic untreated mice were represented through the interrupted line on each panel. Significant concentration differences between treated vs untreated septic mice were indicated through triangles above the respective treatment column, whereas significant differences between treatment options were indicated through stars above connecting lines. The fold decreases underneath each panel reflect the chemokine decreases in response to combined (GENT and PTX) as compared to untreated sepsis. Chemokine (C-X-C motif) ligand 1 (CXCL-1), chemokine (C-C motif) ligand 2 (CCL2), (*) noticeable but non-significant difference p≥0.05, *p<0.05, **p<0.01, ***p<0.001, Δ p<0.05, ΔΔ p<0.01, ΔΔΔ p<0.001. **(C)**: p = 0.075 for (*), **(D)**: p = 0.087 for (*).

*E. coli* sepsis induced a robust expression of all measured chemokines in renal tissue (**Figure 5**). GENT alone as well as (GENT and PTX) significantly decreased C-X-C motif chemokine ligand 1 (CXCL-1), chemokine (C-C motif) ligand 2 (**CCL2)**, and CCL3, and (GENT and PTX) also significantly diminished CCL4 concentrations in renal tissue compared to untreated septic mice pups. PTX alone significantly diminished CCL2, CCL3 and CCL4 tissue concentrations. Compared to GENT alone, (GENT and PTX) led to a significant further decrease in renal tissue concentrations of CCL2 and CCL4, with a non-significant trend towards lower CCL3 concentrations in renal tissue of combined (GENT and PTX) *vs* GENT treatment alone. Taking the fact that chemokines attract neutrophils and other inflammatory cells into the tissue into account, the profound chemokine suppressing effects of (GENT and PTX), ranging from a 5.8-fold decrease for CCL2 to a 42.9-fold decrease for CCL3 compared to renal tissue from untreated septic mice pups may be of particular relevance in diminishing the sepsis-induced tissue damage.

### 3.4 Sex differentially affects the cytokine and chemokine treatment responses to GENT, PTX, and combined (GENT and PTX) in *E. coli*-septic newborn mice

**Table 1** demonstrates the differential anti-inflammatory treatment responses in blood plasma and homogenized renal tissue supernatants of *E. coli-* septic neonatal pups within each sex cohort based on linear mixed effects models with adjustment of the interaction term (sex*treatment). The TNF-inhibiting effects of GENT, PTX and combined (GENT and PTX) in plasma were only observed in female mice, whereas male pups increased TNF concentrations in renal tissue in response to combined (GENT and PTX) vs GENT or PTX alone (See also **Figure 4**). By contrast, the GENT-induced inhibition of renal tissue and plasma IL-1β concentration was only present within the cohort of male pups. Suppression of IL-6 expression in plasma after GENT and (GENT and PTX) compared to PTX alone was observed among female and male pups, and compared to untreated sepsis only among female neonatal mice. Renal IL-6 concentrations were reduced in response to GENT- and (GENT combined with PTX) compared to PTX alone in the male group, whereas PTX alone actually increased IL-6 in renal tissue and plasma of male mice. While PTX alone or in combination with GENT enhanced anti-inflammatory IL-10 plasma concentrations compared to GENT alone, and PTX increased IL-10 compared to untreated sepsis among the entire group of *E. coli*-septic neonatal mice, only male mice demonstrated an increase in plasma IL-10 after PTX alone. Therefore, female and male pups with *E. coli* sepsis demonstrated a differential pattern of responses to these antibiotic and anti-inflammatory treatment regimens, with opposing effects of cytokine expression with some combinations, an observation that may have translational implications.

**Table 1:** Pairwise comparison of renal tissue and plasma cytokines between treatment groups within each sex cohort based on linear mixed effect models with adjustment of the interaction term (sex*treatment).

Analyte	Female	Male	Analyte	Female	Male
Kidney	Treatment group	Treatment group	Plasma	Treatment group	Treatment group
**IL-1β**	G+P ≈ GENT	G+P > GENT***	**IL-1β**	G+P ≈ GENT	G+P > GENT*
	G+P ≈ PTX	G+P ≈ PTX		G+P ≈ PTX	G+P ≈ PTX
	G+P ≈ SAL	G+P ≈ SAL		G+P ≈ SAL	G+P ≈ SAL
	GENT ≈ PTX	GENT < PTX***		GENT ≈ PTX	GENT < PTX***
	GENT ≈ SAL	GENT < SAL**		GENT ≈ SAL	GENT ≈ SAL
	PTX ≈ SAL	PTX ≈ SAL		PTX ≈ SAL	PTX > SAL**
**IL-6**	G+P ≈ GENT	G+P ≈ GENT	**IL-6**	G+P ≈ GENT	G+P ≈ GENT
	G+P ≈ PTX	G+P < PTX*		G+P < PTX**	G+P < PTX*
	G+P ≈ SAL	G+P ≈ SAL		G+P < SAL***	G+P ≈ SAL
	GENT ≈ PTX	GENT < PTX*		GENT < PTX**	GENT < PTX**
	GENT ≈ SAL	GENT ≈ SAL		GENT < SAL**	GENT ≈ SAL
	PTX ≈ SAL	PTX > SAL*		PTX ≈ SAL	PTX > SAL*
**IL-10**	G+P ≈ GENT	G+P ≈ GENT	**IL-10**	G+P ≈ GENT	G+P ≈ GENT
	G+P ≈ PTX	G+P ≈ PTX		G+P ≈ PTX	G+P ≈ PTX
	G+P ≈ SAL	G+P ≈ SAL		G+P ≈ SAL	G+P ≈ SAL
	GENT ≈ PTX	GENT ≈ PTX		GENT ≈ PTX	GENT ≈ PTX
	GENT ≈ SAL	GENT ≈ SAL		GENT ≈ SAL	GENT ≈ SAL
	PTX ≈ SAL	PTX ≈ SAL		PTX ≈ SAL	PTX > SAL**
**TNF**	G+P ≈ GENT	G+P > GENT*	**TNF**	GENT < SAL*	G+P ≈ GENT
	G+P ≈ PTX	G+P > PTX*		G+P ≈ GENT	G+P ≈ PTX
	G+P ≈ SAL	G+P ≈ SAL		G+P ≈ PTX	G+P ≈ SAL
	GENT ≈ PTX	GENT ≈ PTX		G+P < SAL***	GENT ≈ PTX
	GENT ≈ SAL	GENT ≈ SAL		GENT ≈ PTX	GENT ≈ SAL
	PTX ≈ SAL	PTX ≈ SAL		PTX < SAL**	PTX ≈ SAL

> indicates that the first treatment group showed higher cytokine concentrations compared to the second treatment of the respective comparison.

**<** indicates that the first treatment group showed lower cytokine concentrations compared to the second treatment of the respective comparison.

≈ indicates no significant difference between treatment groups.

P-values were derived from t-tests based on linear mixed effect models. Significant comparisons were indicated in bold, and significance levels were represented by asterisks: *p<0.05, **p<0.01, ***p<0.001.

Diminished IL-1α, IL-12 (p40), and IL-17 renal tissue concentrations after combined (GENT and PTX) were primarily observed in our female neonatal mice cohort, whereas inhibition of expression of these same cytokines among male pups occurred primarily after GENT exposure (**Supplementary Table S1**). G-CSF renal tissue concentrations were inhibited in response to GENT or (GENT and PTX) among female and male cohorts of pups (**Supplementary Table S1**).

Renal tissue chemokine concentrations of CXCL-1, CCL2, CCL3, and CCL4 (**Table 2**), on the other hand, mostly demonstrated comparable patterns of suppression after GENT and (GENT and PTX) treatment among the male and female cohorts.

**Table 2:** Pairwise comparison of renal tissue chemokines between treatment groups within each sex cohort based on linear mixed effect models with adjustment of the interaction term (sex*treatment).

Analyte	Female	Male
Kidney	Treatment group	Treatment group
**CXCL-1**	G+P ≈ GENT	G+P > GENT*
	G+P < PTX**	G+P < PTX***
	G+P < SAL***	G+P < SAL**
	GENT < PTX**	GENT < PTX***
	GENT < SAL***	GENT < SAL***
	PTX ≈ SAL	PTX ≈ SAL
**CCL2**	G+P < GENT**	G+P ≈ GENT
	G+P ≈ PTX	G+P ≈ PTX
	G+P < SAL***	G+P < SAL**
	GENT ≈ PTX	GENT ≈ PTX
	GENT ≈ SAL	GENT < SAL*
	PTX < SAL**	PTX ≈ SAL
**CCL3**	G+P < GENT*	G+P ≈ GENT
	G+P < PTX**	G+P < PTX***
	G+P < SAL***	G+P < SAL***
	GENT ≈ PTX	GENT < PTX***
	GENT < SAL***	GENT < SAL***
	PTX < SAL**	PTX ≈ SAL
**CCL4**	G+P < GENT*	G+P ≈ GENT
	G+P ≈ PTX	G+P ≈ PTX
	G+P < SAL***	G+P < SAL*
	GENT ≈ PTX	GENT ≈ PTX
	GENT ≈ SAL	GENT < SAL*
	PTX < SAL*	PTX ≈ SAL

> indicates that the first treatment group showed higher chemokine concentrations compared to the second treatment of the respective comparison.

**<** indicates that the first treatment group showed lower chemokine concentrations compared to the second treatment of the respective comparison.

≈ indicates no significant difference between treatment groups.

P-values were derived from t-tests based on linear mixed effect models. Significant comparisons were indicated in bold, and significance levels were represented by asterisks: *p<0.05, **p<0.01, ***p<0.001.

### 3.5 GENT combined with PTX mitigated the expression of biomarkers of kidney injury in *E. coli*-septic newborn mice

**Figure 6:**
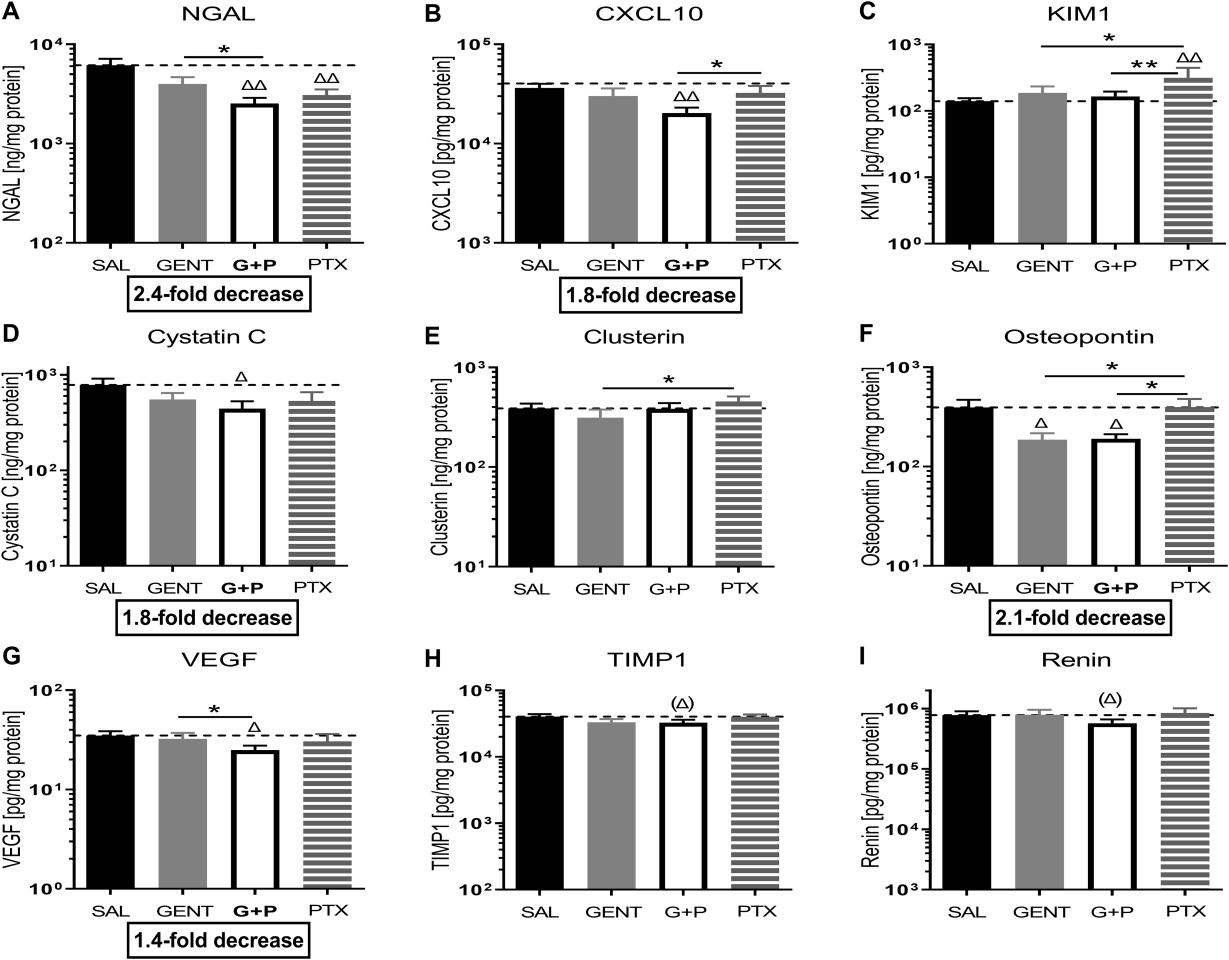
Combined GENT and PTX mitigate the expression of biomarkers of kidney injury in *E. coli*-septic newborn mice. Neonatal mice pups less than 24 hours old were injected iv with live 10^5^ CFU *E. coli* per g body weight, and treated with GENT (n = 18), PTX (n = 23), combined (GENT and PTX [G+P], n = 24), or left untreated (SAL, n = 33) 1.5 hours after bacterial injections and observed for further 4 hours. Kidney injury biomarker concentrations measured in renal tissue lysate supernatants for **(A)** NGAL, **(B)** CXCL-10, **(C)** KIM-1, **(D)** cystatin C, **(E)** clusterin, **(F)** osteopontin, **(G)** VEGF, **(H)** TIMP-1, and **(I)** renin are shown. Mean biomarker concentrations in septic untreated mice were represented through the interrupted line on each panel. Significant concentration differences between treated vs untreated septic mice were indicated through triangles above the respective treatment column, whereas significant differences between treatment options were indicated through stars above connecting lines. The fold decreases underneath panels **(A, B, D, F, G)** reflect the biomarker decreases in response to combined (GENT and PTX) as compared to untreated sepsis. Chemokine (C-X-C motif) ligand 10 (CXCL10), vascular endothelial growth factor (VEGF), tissue inhibitor of metalloproteinases 1 (TIMP-1), *p<0.05, **p<0.01, Δ p<0.05, ΔΔ p<0.01, (Δ) noticeable but non-significant difference p≥0.05. **(H)**: p= 0.083 for (Δ), **(I)**: p=0.12 for (Δ).

As presented in **Figure 6**, addition of PTX to GENT, but not GENT alone significantly suppressed the expression of several biomarkers of kidney injury in renal tissue lysates of *E. coli*-septic newborn mice, including NGAL (p<0.01), CXCL-10 (p<0.01), cystatin C (p<0.05), and VEGF (p<0.05). GENT as well as (GENT and PTX) diminished renal tissue expression of osteopontin (p < 0.05). TIMP-1 (p=0.083) and renin (p=0.12) showed a non-significant decrease in response to combined (GENT and PTX) treatment compared to untreated sepsis. Furthermore, (GENT and PTX) resulted in significantly lower concentrations of NGAL and VEGF in renal tissue homogenate supernatants of these mice compared to GENT alone, and to lower concentrations of CXCL-10, KIM-1, and osteopontin compared to PTX. KIM-1 concentrations were however not diminished with combined (GENT and PTX) compared to untreated *E. coli* sepsis, and PTX alone even further increased renal tissue KIM-1 expression.

When analyzing these biomarkers of kidney injury separately among female and male cohorts of septic pups, diminished biomarker concentrations in response to treatment were primarily observed in female newborn mice (NGAL, CXCL10, KIM-1, VEGF, tissue inhibitor of metalloproteinase 1 (TIMP-1), and osteopontin) (**Table 3**; **Supplementary Table S2**). Among male mice, combined (GENT and PTX) resulted in significantly reduced NGAL renal tissue concentrations (**Table 3**), whereas GENT alone diminished cystatin C and osteopontin compared to untreated septic male mice (**Supplementary Table S2**). The observed average TIMP-1 and KIM-1 concentrations for the entire cohort of mice and the lack of concentration changes with (GENT and PTX) exposure (**Figure 6**) could therefore be explained by sex differences in the expression of these renal injury markers.

**Table 3:** Pairwise comparison of biomarkers of renal injury between treatment groups within each sex cohort based on linear mixed effect models with adjustment of the interaction term (sex*treatment).

Analyte	Female	Male
Kidney	Treatment group	Treatment group
**KIM-1**	G+P ≈ GENT	G+P ≈ GENT
	G+P < PTX**	G+P ≈ PTX
	G+P ≈ SAL	G+P ≈ SAL
	GENT < PTX*	GENT ≈ PTX
	GENT ≈ SAL	GENT ≈ SAL
	PTX > SAL**	PTX ≈ SAL
**CXCL10**	G+P < GENT*	G+P ≈ GENT
	G+P ≈ PTX	G+P ≈ PTX
	G+P < SAL**	G+P ≈ SAL
	GENT ≈ PTX	GENT ≈ PTX
	GENT ≈ SAL	GENT ≈ SAL
	PTX ≈ SAL	PTX ≈ SAL
**VEGF**	G+P < GENT*	G+P ≈ GENT
	G+P ≈ PTX	G+P ≈ PTX
	G+P ≈ SAL	G+P ≈ SAL
	GENT ≈ PTX	GENT ≈ PTX
	GENT ≈ SAL	GENT ≈ SAL
	PTX ≈ SAL	PTX ≈ SAL
**NGAL**	G+P < GENT*	G+P ≈ GENT
	G+P ≈ PTX	G+P ≈ PTX
	G+P < SAL*	G+P < SAL*
	GENT ≈ PTX	GENT ≈ PTX
	GENT ≈ SAL	GENT ≈ SAL
	PTX < SAL*	PTX ≈ SAL

> indicates that the first treatment group showed higher kidney injury biomarker concentrations compared to the second treatment of the respective comparison.

**<** indicates that the first treatment group showed lower kidney injury biomarker concentrations compared to the second treatment of the respective comparison.

≈ indicates no significant difference between treatment groups.

P-values were derived from t-tests based on linear mixed effect models. Significant comparisons were indicated in **bold**, and significance levels were represented by asterisks: *p<0.05, **p<0.01.

### 3.6 CEF with or without PTX inhibited *E. coli*-induced chemokine expression and enhanced IL-10 in renal tissue of septic neonatal mice

**Figure 7:**
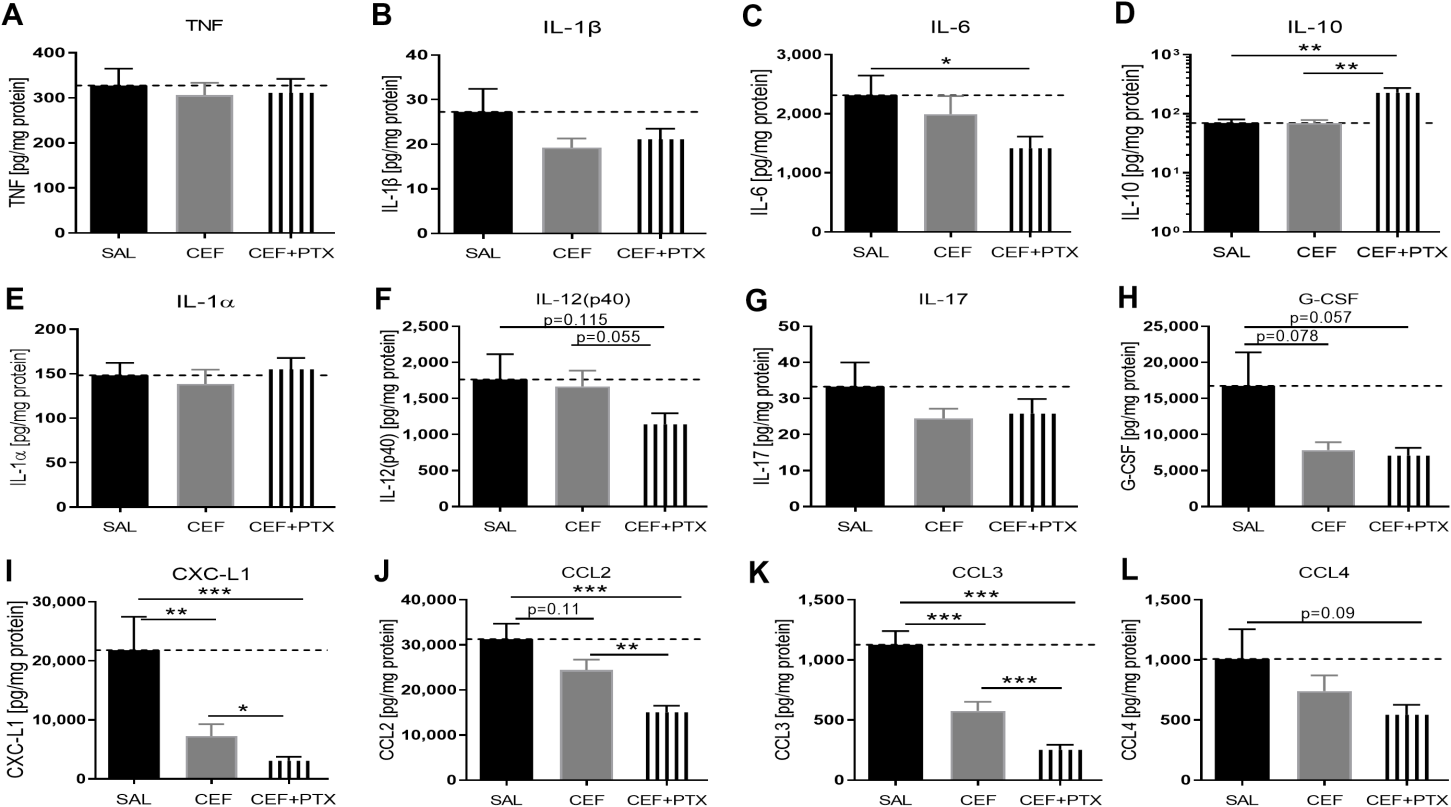
CEF with or without PTX inhibit *E. coli*-induced chemokine expression and enhance IL-10 in renal tissue of septic neonatal mice. Pups less than 24 hours old were injected ip with live 10^5^ CFU *E. coli* per g body weight, and treated with CEF (n = 24), combined (CEF and PTX, n = 23), or left untreated (SAL, n = 18) 2 hours after bacterial injections and observed for further 4 hours. Cytokine and chemokine concentrations were measured in renal tissue lysate supernatants for **(A)** TNF, **(B)** IL-1β, **(C)** IL-6, **(D)** IL-10, **(E)** IL-1α, **(F)** IL-12 (p40), **(G)** IL-17, **(H)** G-CSF, **(I)** CXCL1, **(J)** CCL2, **(K)** CCL3, **(L)** CCL4. Mean cytokine and chemokine concentrations in septic untreated mice were represented through the interrupted line on each panel. Significant concentration differences between treatment groups were indicated through stars above connecting lines. *p<0.05, **p<0.01, ***p<0.001.

Due to the known nephrotoxicity of GENT, albeit of low risk in these short-duration experiments of sepsis-induced AKI, we conducted similar experiments with CEF as antimicrobial agent. Sepsis in neonatal mice was induced through ip injection of *E. coli* K1 strain (10^5^ CFU per g body weight). 2 hours after the injection of bacteria, mice were randomly selected for injection of CEF, (CEF and PTX), or an equal volume of sterile saline (representing untreated sepsis). After an additional 4 hours of observation, mice were euthanized and renal tissues were harvested, homogenized, and cytokines and chemokines were measured in supernatants of kidney lysates. Of note, all mice in this experiment including untreated septic pups survived the observation period. Cytokine concentration changes measured after CEF or (CEF and PTX) exposure (**Figure 7**) were comparable to those with GENT. (CEF and PTX) combined but not CEF alone decreased IL-6 tissue concentrations compared to untreated sepsis mouse pups. Addition of PTX to CEF significantly increased the anti-inflammatory IL-10 expression in renal tissue lysates compared to untreated (1.85-fold increase) as well as CEF-treated septic pups (p<0.01). No changes were however observed in the renal tissue concentrations of TNF and IL-1β, which may be due to sex differences in immune responses and/or the timing of the inflammatory response. IL-12 (p40) and G-CSF showed only a non-significant trend towards diminished cytokine concentrations in response to (CEF and PTX) exposure compared to untreated septic mice [IL-12 (p40), G-CSF], and compared to CEF alone [IL12 (p40)], whereas IL-1α and IL-17 did not show any differences in their renal tissue concentrations.

As with (GENT and PTX), chemokine concentrations in renal tissues were markedly suppressed after combined administration of (CEF and PTX) to septic neonatal pups, with 7-fold decrease compared to untreated septic pups for CXCL-1, 2-fold decrease for CCL2, and 4.5-fold decrease for CCL3. Furthermore, (CEF and PTX) significantly inhibited renal tissue expression of CXCL-1, CCL2, and CCL3 compared to CEF alone, and CEF on the other hand significantly diminished CXCL-1 and CCL3 compared to untreated septic mice, with a non-significant trend towards lower CCL2 concentrations with CEF compared to untreated septic neonatal mice.

### 3.7 Effect of combined CEF and PTX on renal tissue injury biomarker expression in neonatal mice with *E. coli* sepsis

**Figure 8:**
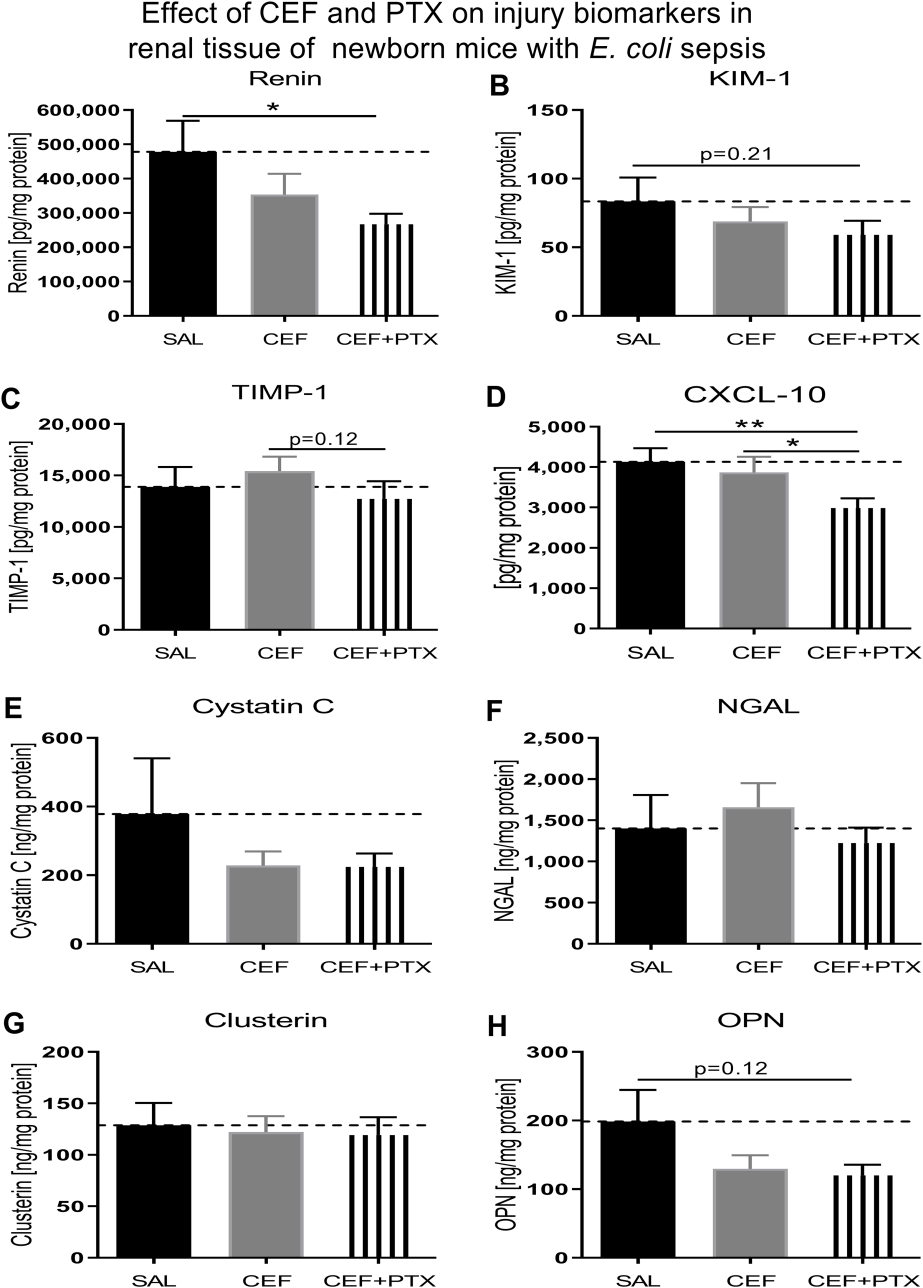
Addition of PTX to CEF reduces the expression of biomarkers of renal injury in tissue lysates of kidneys obtained from neonatal *E. coli*-septic mice. Newborn mice less than 24 hours old were injected ip with live 10^5^ CFU *E. coli* per g body weight, and treated with CEF (n = 24), combined (CEF and PTX, n = 23), or left untreated (SAL, n = 18) 2 hours after bacterial injections and observed for further 4 hours. Kidney injury biomarker concentrations were measured in renal tissue lysates for **(A)** renin, **(B)** KIM-1, **(C)** TIMP-1, **(D)** CXCL-10, **(E)** cystatin C, **(F)** NGAL, **(G)** clusterin, and **(H)** osteopontin (OPN). Mean biomarker concentrations in septic untreated mice were represented through the interrupted line on each panel. Significant concentration differences between treatment groups were indicated through stars above connecting lines. *p<0.05, **p<0.01.

Combined exposure to (CEF and PTX) diminished the expression of renal injury biomarkers in kidneys of our septic neonatal mice compared to untreated mice (**Figure 8**). These include renin and CXCL10, whereby (CEF and PTX) also decreased the renal tissue concentration of CXCL10 compared to CEF alone. Non-significant trends of lower renal tissue concentrations after (CEF and PTX) exposure compared to untreated septic neonatal mice were observed for KIM-1, TIMP-1, and osteopontin. As opposed to the findings after (GENT and PTX) exposure, NGAL concentrations were not diminished following treatment with CEF or (CEF and PTX).

### 3.8 Effects of murine neonatal *E. coli* sepsis on renal histology

**Figure 9:**
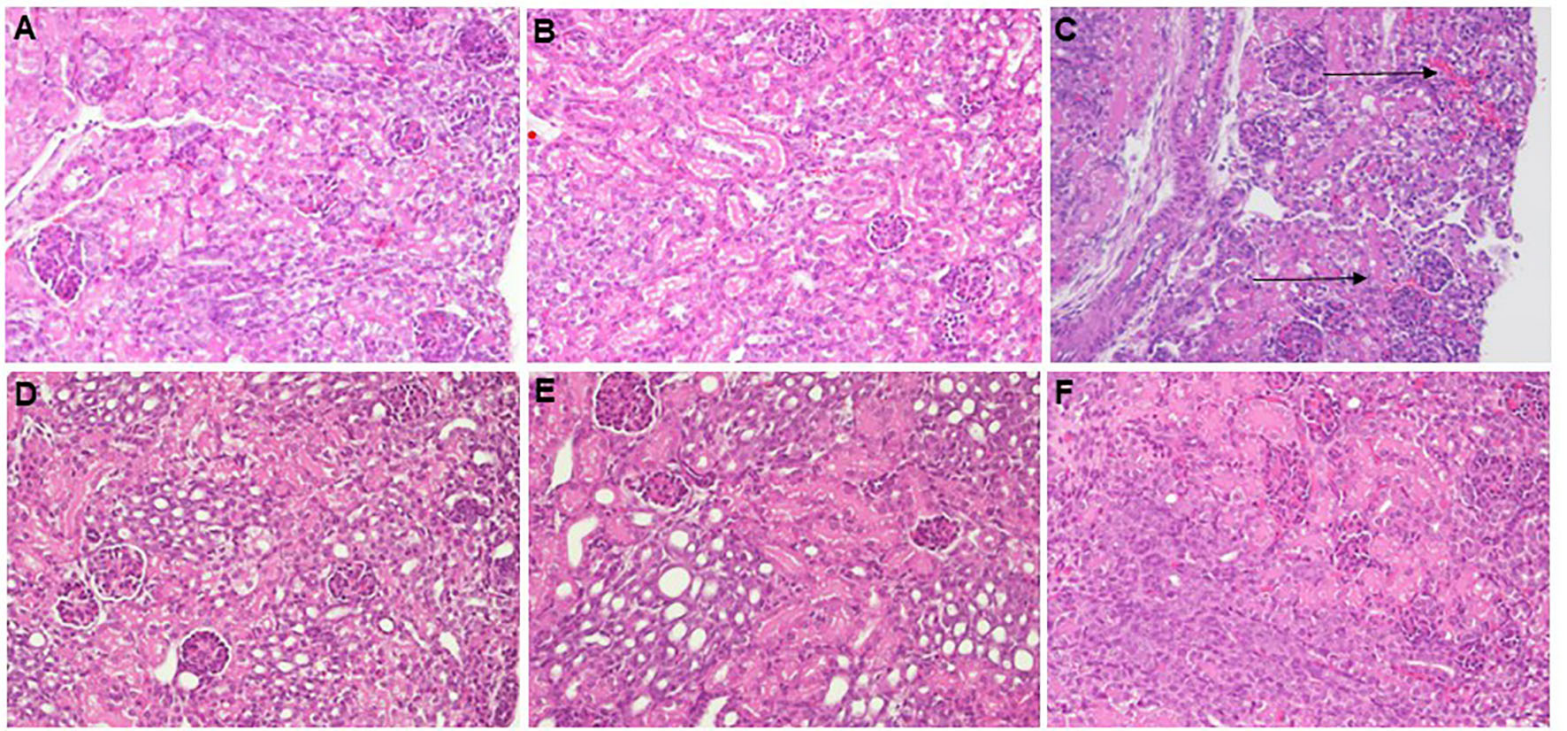
Histological imaging of kidneys from healthy controls and *E. coli*-septic mice. Shown are representative PAS- **(A, B, F)** and H&E-stained **(C–E)** images at 20x magnification of paraffin-embedded and formalin-fixed kidney sections, derived from 7-days old C57BL/6J healthy controls **(A)**, untreated *E. coli-*septic mice **(B, C)**, and *E. coli*-septic mice treated with CEF **(D)** and combined CEF and PTX (**Figure 9**) **(E, F)** 12 hours after ip injection of *E. coli* (10^5^ CFU/g body weight). Renal sections from CEF-, CEF and PTX-, as well as untreated *E. coli-*septic mice appeared mostly normal with intact tubular epithelium and brush border, intact glomeruli, and without interstitial inflammation or tubulointerstitial scaring, whereas several kidneys from septic animals revealed focal areas of glomerular congestion and interstitial hemorrhage **(C)**, indicated by arrows), likely representing shock kidneys.

**Figure 9** shows representative H&E- (**Figures 9C–E** *)* and PAS-stained (**Figures 9A, B, F** *)* images of paraffin-embedded and formalin-fixed kidney sections, derived from 7-days old C57BL/6J healthy controls (**Figure 9A**), untreated *E. coli-*septic mice (**Figures 9B, C** *)*, and *E. coli*-septic mice treated with CEF (**Figure 9D**) and combined CEF and PTX (**Figures 9E, F** *)* 12 hours after ip injection of *E. coli* (10^5^ CFU/g body weight). Of note, 3 pups (30%) of untreated septic mice in this experiment did not survive the 12-hour observation period, and were excluded from histological analysis due to postmortem changes. PAS reacts with carbohydrates present in the brush border of renal tubular cells as well as basement membranes of glomerular capillaries and tubular epithelia, and can therefore be used to identify potential loss or damage to the proximal tubule brush border. Whereas renal sections from *E. coli-*septic 7-days old mice appeared mostly normal, several kidneys from septic animals revealed focal areas of glomerular congestion and interstitial hemorrhage (**Figure 9C** *)*, likely representing shock kidneys.

### 3.9 Immunofluorescence imaging of kidney injury markers in *E. coli*-septic neonatal mice *vs* controls

**Figure 10:**
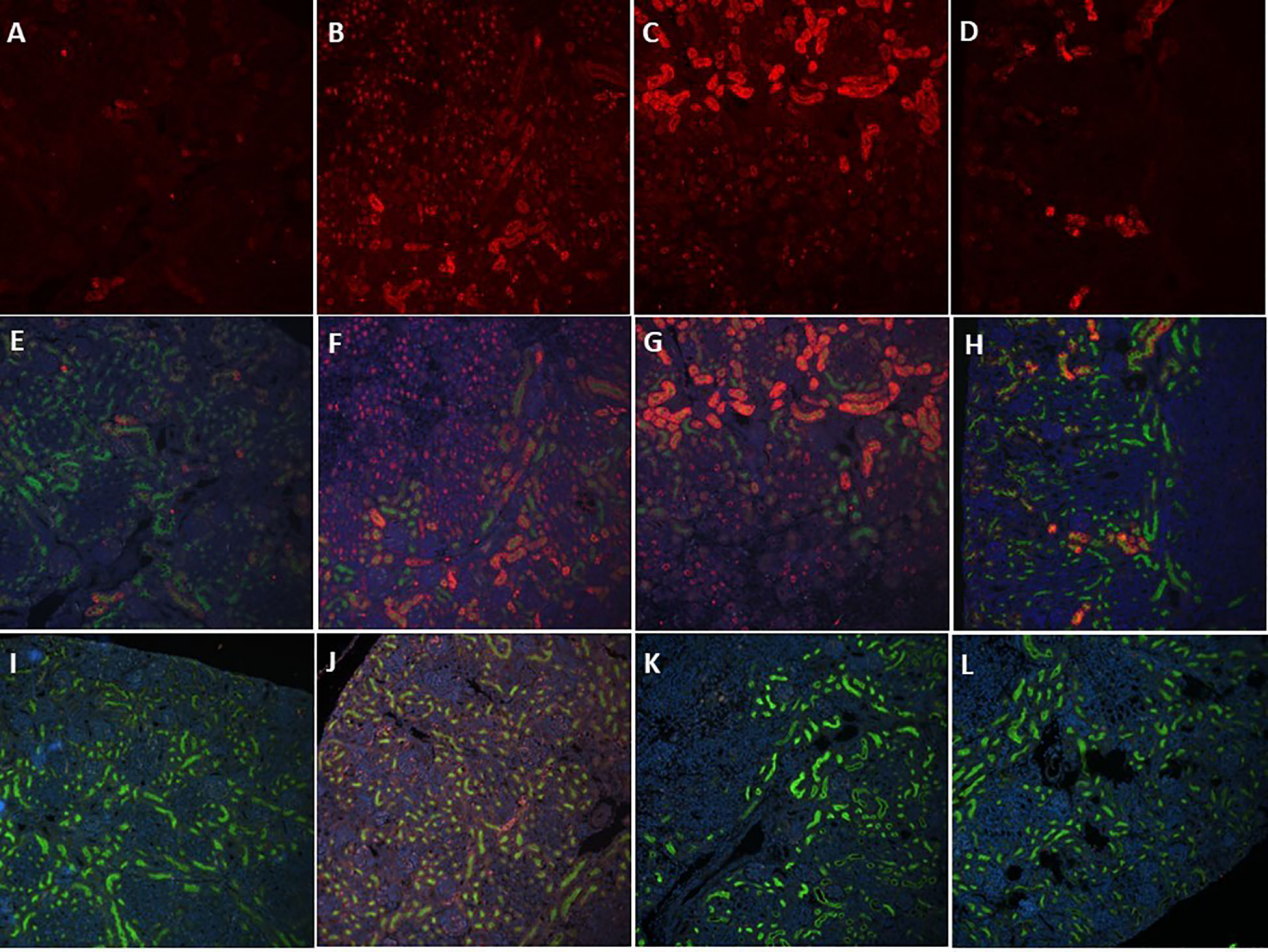
Increased NGAL- and KIM-1 staining of proximal tubules in *E. coli*-septic newborn mice. Immunofluorescent images of formalin-fixed paraffin-embedded kidney sections from 7 days old healthy controls **(A, E, I)**, untreated *E. coli*-septic pups **(B, F, J)**, as well as *E. coli*-septic pups treated with CEF **(C, G, K)** or combined CEF and PTX **(D, H, L)** 12h after bacterial ip injection (*E. coli* 10^5^ CFU/g body weight) after anti-NGAL (cat. # ab216462, *Abcam; Waltham, MA*) **(A-H)** or anti-KIM-1 (cat. # AF1817, *R&D Systems; Minneapolis, MN*) **(I–L)** labeling, Alexa Fluor 568-conjugated secondary antibody, Hoechst (*AnaSpec, Inc.; Fremont, CA*), and fluorescein-conjugated *Lotus Tetragonolobus* lectin (*Vector Laboratories, Inc.; Newark, CA*) staining, at 20x magnification. Panels **(A–D)** show the red channel only (NGAL), panels **(E–H)** represent 3-color overlays for NGAL-stained, and panels **(I–L)** demonstrate 3-channel overlays of KIM-1-stained images (red: NGAL or KIM-1, respectively; green: *Lotus Tetragonolobus* lectin; blue: DAPI). These representative images demonstrated increased staining for the injury markers NGAL and KIM-1 in septic pups compared to controls (see also **Figure 11**), as well as the effects of antibiotic and anti-inflammatory treatment with CEF with/without PTX.

**Supplementary Figure S2** describes our quantification method of fluorescent immunostaining of kidney injury markers from whole renal scans (**Supplementary Figure S2A**) with ImageJ. As illustrated in **Supplementary Figure S2B**, regions of interest **(ROIs)** were manually placed to outline the renal cortex, excluding any larger cystic structures if present, followed by 3-color image acquisition [red: KIM-1 or NGAL, respectively; green: *Lotus Tetragonolobus* lectin; blue: 4′,6-diamidino-2-phenylindole (DAPI)], and fluorescence intensity and area measurements within ROIs using ImageJ software. As indicated in *Supplemental Panel 2C*, septic mice showed low (arrow 1), medium (arrow 2) and high (arrow 3) intensity NGAL-staining of tubules, whereas control mice demonstrated primarily low intensity NGAL-staining (not shown). Consequently, fluorescence intensity measurements were better able to differentiate NGAL expression between septic and control pups as opposed to areas of fluorescence above threshold, whereas area of fluorescence measurements were employed for the quantification of KIM-1 labeling. **Figure 10** demonstrates representative immunofluorescence images of kidney sections from 7-days old control (**Figures 10C, E, I** *)*, untreated *E. coli*-septic pups (**Figures 10B, F, J** *)*, as well as *E. coli*-septic pups treated with CEF (**Figures 10C, G, K** *)* or combined CEF and PTX (**Figures 10D, H, L** *)* 12h after bacterial ip injection after anti-NGAL (**Figures 10A–H**) or anti-KIM-1 (**Figures 10I–L**) labeling. These representative images demonstrated increased staining for the renal injury markers KIM-1 and NGAL in septic pups compared to healthy controls, as well as diminished NGAL expression after combined CEF and PTX exposure, and decreased KIM-1 fluorescent staining after CEF or combined CEF and PTX. Whereas most controls and septic mice treated with CEF or combined CEF and PTX showed no tubular anti-KIM-1 staining, there were several outliers present, as shown in **Supplementary Figure S3**. The control pup in *panel (A)*, as well as the CEF-treated mouse in *panel (B)* and the pup exposed to combined CEF and PTX in *panel (C)* represented outliers with moderate degrees of anti-KIM-1 staining.

### 3.10 Increased renal cortical NGAL and KIM-1 staining in *E. coli*-septic neonatal mice

**Figure 11:**
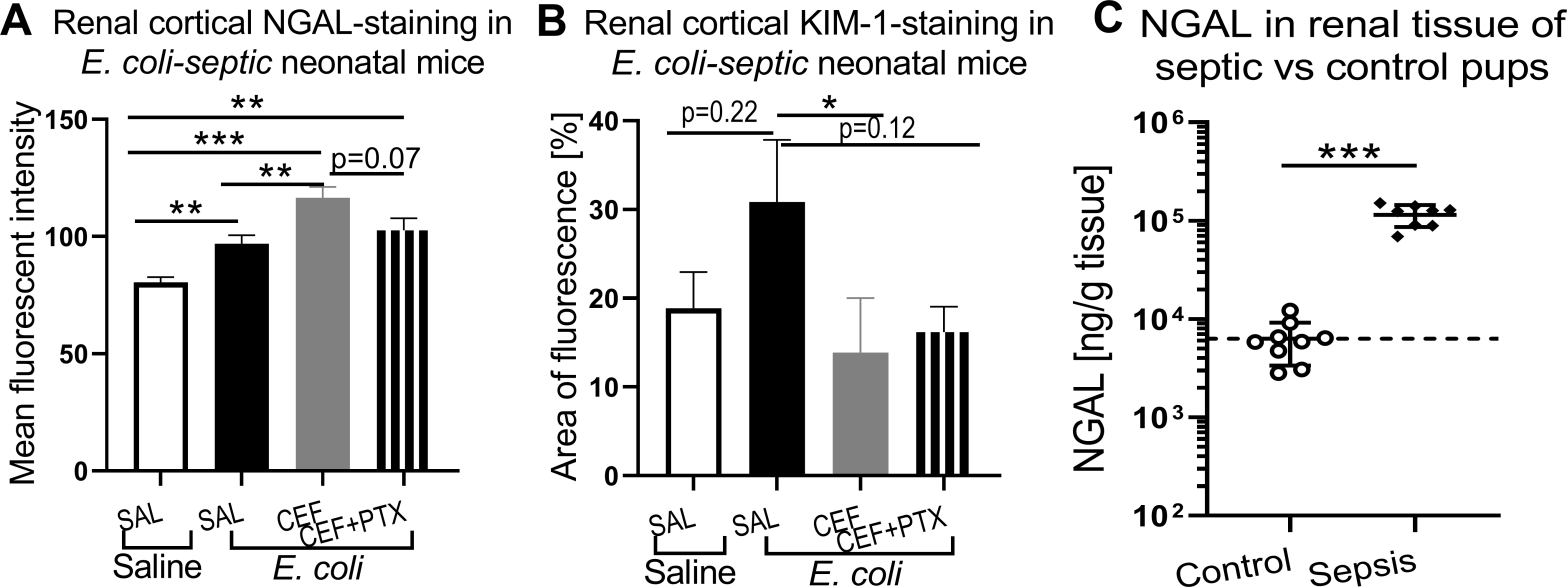
Effects of CEF alone or combined with PTX on renal cortical NGAL and KIM-1 staining among *E. coli*-septic pups. Immunofluorescent images of kidney sections from 7 days old *E. coli*-septic pups 12h after bacterial ip injection (*E. coli* 10_5_ CFU/g body weight), that were treated or not with CEF alone or combined with PTX vs healthy controls, were quantitatively analyzed employing ImageJ software. Regions of interest (ROIs) were manually placed onto images from whole renal scans to outline the renal cortex, followed by 3-channel image acquisition (red: KIM-1 or NGAL, respectively; green: *Lotus Tetragonolobus* lectin; blue: DAPI), and measurements of fluorescence intensity for NGAL **(A)** and area of fluorescence above threshold **(B)** for KIM-1 within ROIs using ImageJ software. For comparison, (**C**) demonstrates NGAL concentrations measured from renal tissue lysates of uninfected vs *E. coli*-septic postnatal day 1 pups. **(A)** and **(B)**: uninfected controls (n=5), untreated septic pups (n = 6), CEF-treated pups (n = 7), combined CEF+PTX (n = 8). Significant differences between treatment options were indicated through stars above connecting lines: *p<0.05, **P<0.01, ***p<0.001. Mean chemokine concentrations in uninfected control mice in **(C)** were represented through the interrupted line.

**Figure 11** shows the findings from the quantitative analysis of NGAL and KIM-1 staining within the renal cortex as outlined by ROIs. **Figure 11A** demonstrated significantly higher mean fluorescent intensity for NGAL among untreated *E. coli*-septic mice compared to uninfected controls (p<0.01), which is consistent with the significantly higher renal tissue NGAL concentrations among postnatal day 1 *E. coli*-septic *vs* control pups (p<0.001) (**Figure 11C**). CEF alone further significantly increased NGAL fluorescent intensity compared to kidneys from untreated septic mice (p<0.01), while addition of PTX to CEF mitigated the antimicrobial treatment-induced increases in NGAL-staining (p=0.07 for combined CEF and PTX *vs* CEF alone). KIM-1 (**Figure 11B**), on the other hand, indicated a non-significant trend (p=0.22) towards an increased area of fluorescence above threshold of renal sections from untreated septic mice compared to controls, whereas CEF alone suppressed *E. coli-*induced KIM-1 expression (p<0.05), with a non-significant decrease in KIM-1 area of fluorescence for combined CEF and PTX (p=0.12, n = 6-8). Based on these findings we concluded that neonatal sepsis led to increased expression of kidney injury markers, which significantly improved with CEF and PTX treatment.

## 4 Discussion

Neonatal sepsis is one of the most common risk factors for AKI in neonates, further increasing its mortality and potentially leading to long-term renal dysfunction among survivors (Abitbol et al., 2003), for which no proven effective treatments are available. To address this problem, we developed a model of murine neonatal sepsis-induced AKI of mice less than 24 hours old, i.e., during a developmental stage of ongoing nephrogenesis and immature immunity comparable to that of human preterm septic newborns (Adkins et al., 2004; Stelloh et al., 2012; Saint-Faust et al., 2014; Carpenter et al., 2023). As demonstrated through our studies presented here, where we administered antibiotics with or without PTX, this model is suitable to study the effects of candidate pharmacological agents for the treatment of early life bacterial sepsis and AKI.

When we applied our neonatal *E. coli* sepsis model, we measured high concentrations of cytokines and chemokines in renal tissue lysates and in plasma, which was accompanied by high bacterial CFUs in tissue and blood. Treatment with either gentamicin or cefotaxime alone or in combination with PTX significantly inhibited a number of inflammatory cytokines (such as IL-1β and IL-6) and profoundly suppressed the expression of chemokines in renal tissue (CXCL-1, CCL2, CCL3, and CCL4). These chemokines subsequently attract neutrophils and other innate and adaptive immunity effector cells to the tissue, along with induction of proliferation, differentiation, cytokine production, cellular degranulation and respiratory burst, thus initiating a whole cascade of events (Hughes and Nibbs, 2018). The marked inhibition of renal tissue chemokines with combined antibiotic and PTX exposure may therefore have profound effects on reduction of local inflammation and the integrity of the tissue.

Systemic cytokine concentrations and their modifications in response to antibiotic and/or PTX exposure differed from those measured in renal tissue homogenates in our current study, and similar findings have previously been reported by us for other peripheral organs (lung, liver, spleen, brain) (Speer et al., 2020). Sustained inflammation due to sepsis may lead to organ injury including the brain and kidney (Cavaillon et al., 2014; Strunk et al., 2014), suggesting that targeting excessive and/or sustained inflammation accompanying sepsis through appropriately timed anti-inflammatory agents may be beneficial.

Our studies in septic neonatal mice revealed high concentrations of biomarkers of kidney injury, as measured in renal tissue lysates or through immunofluorescence imaging. A variety of these markers of kidney injury were significantly reduced in response to combined antibiotic (GENT or CEF) and PTX treatment, notably NGAL, CXCL-10, cystatin C, osteopontin, and VEGF after combined GENT and PTX exposure. Furthermore, diminished CXCL-10 and renin, as well as non-significant trends towards decreased biomarker concentrations for KIM-1, TIMP-1, and osteopontin were noted after CEF and PTX treatment, suggesting that addition of PTX to antibiotics may reduce acute kidney injury during neonatal sepsis. These results are in line with studies in adult rodents, where PTX has demonstrated renal protection from ischemia and endotoxinemia (Wang et al., 2006; Bansal et al., 2009; Wystrychowski et al., 2014), as well as diabetic kidney disease (Navarro-González et al., 2015). Besides its anti-inflammatory activity (Speer et al., 2017; Speer et al., 2018), PTX promoted mitochondrial biogenesis and integrity, and demonstrated antioxidant effects in the brain (Khedr et al., 2019; Wang et al., 2020; Wang et al., 2021), all of which can be beneficial in the context of sepsis. PTX reduced the expression of kidney injury biomarkers in combination with either GENT or CEF, despite the known nephrotoxicity of GENT. This might be due to the limited GENT exposure in our short-duration experiment, or alternatively, because of its counteracting nephroprotection (Stojiljkovic et al., 2009; Nasiri-Toosi et al., 2013). We have previously demonstrated synergistic anti-inflammatory effects in human cord blood samples exposed to TLR agonists, when combining PTX with the macrolide antibiotic azithromycin (Speer et al., 2018), thus increasing the range of potential candidate antimicrobial agents with which PTX could be combined, pending confirmatory preclinical and clinical studies. PTX might therefore exert nephroprotective actions during sepsis that are independent of the specific antibiotics applied.

Our interaction analysis between animal sex and treatment group revealed that sex differentially affected biomarkers of inflammation and kidney injury in renal tissue as well as blood plasma in response to exposure of PTX, whereby this agent preferentially exerted its anti-inflammatory (e.g., TNF, IL-1α, IL-12(p40), IL-17, and G-CSF) and kidney injury biomarker (including CXCL-10, NGAL, VEGF) reducing (and thus nephroprotective) actions among female pups. Interestingly, suppression of chemokine expression in renal tissue of septic pups treated with combined (GENT and PTX) was comparable between both sexes, indicating that PTX can nevertheless exert potent anti-inflammatory actions in both male and female mice. Gender differences have been reported for sepsis as well as AKI and COVID-19-associated AKI, whereby recent studies mostly demonstrated more favorable outcomes among female patients (Angele et al., 2014; Neugarten et al., 2018; He et al., 2022; Neugarten and Golestaneh, 2022). These reports and our findings of differential treatment effects of PTX by sex may have translational implications in the era of individualized patient medicine. Furthermore, sex differences in treatment responses may have masked the detection of some relevant treatment responses when combining both animal sexes.

Our H&E- and PAS-stained histological images of kidneys from *E. coli*-septic mice pups appeared mostly normal, with the exception of several kidneys from septic animals that demonstrated focal areas of glomerular congestion and interstitial hemorrhage likely representing shock kidneys. As previously reported in a systematic review, there are no consistent histological renal changes in human or animal sepsis-induced AKI, with the majority of studies reporting normal histology or mild nonspecific changes, with acute tubular necrosis being uncommon (Langenberg et al., 2008). Our non-specific histological findings from kidneys affected by neonatal bacterial sepsis are thus compatible with the previously reported sepsis-induced AKI phenotype. The lack of cellular inflammatory infiltration despite high chemokine concentrations in renal tissue of our untreated septic experimental pups is likely due to the time delay in cellular recruitment. Nevertheless, using immunofluorescence staining with specific antibodies directed against NGAL and KIM-1 as well as measurements of these markers in renal tissue homogenates, we were able to show significant increases in the expression of these kidney injury markers among our *E. coli*-septic neonatal mice.

Our study has the following important strengths: We present a novel model of murine neonatal bacterial sepsis-induced AKI, initiated on postnatal day 1 during still ongoing nephrogenesis, making our model more relevant to sepsis-induced AKI in human preterm newborns, the clinical group at highest risk of developing AKI. Our model utilized a virulent strain of *E. coli*, which is a leading pathogen in severe neonatal sepsis. Using live bacteria as opposed to heat-killed microbes or purified TLR agonists provides a more clinically relevant model, considering the immune systems’ ability to distinguish between live or killed bacteria (Sander et al., 2011; Strunk et al., 2011; Mourao-Sa et al., 2013). We employed rigorous quality control measures to assess the adequacy of bacterial injections in our postnatal day 1 mouse pups (Speer et al., 2020). Additionally, we applied linear mixed modeling to control for litter effects and to assess the interaction between treatment and sex, a translationally relevant aspect in view of the divergent clinical trajectories frequently observed among term and preterm neonates of both sexes. By analyzing both systemic and kidney inflammatory cytokines and biomarkers of renal tissue injury and inflammation, our findings on AKI and inflammation were presented in the context of the neonatal host inflammatory sepsis syndrome. Results obtained were comparable between two different antibiotics used, nephrotoxic *vs* non-nephrotoxic, increasing the applicability for PTX treatment to protect against neonatal sepsis-induced AKI. Furthermore, we obtained comparable results when employing two different routes of bacterial administration, namely intravenous and intraperitoneal Gram-negative bacterial injections that model important types of neonatal bacterial sepsis. Despite its greater severity and progression of intravenous bacterial sepsis and the resulting host inflammatory response, combined use of PTX and antibiotics diminished renal tissue inflammation and kidney injury biomarker expression compared to antibiotics alone or untreated sepsis in both murine neonatal sepsis models. As exemplified with our example of combined antibiotic and PTX treatment of septic neonatal mice, our model is suitable for preclinical testing of anti-inflammatory or other pharmacological treatments, thus fulfilling an urgent translational need.

We recognize several limitations of our study. We investigated the acute effects of murine neonatal sepsis-induced AKI and its treatment with PTX in addition to antibiotics. However, prior to implementing this novel approach to the clinic, longitudinal preclinical follow-up studies are required. In addition, the time course of sepsis evolution, inflammation, kidney injury markers, histological and functional renal changes and their modifications in response to these treatment modalities need to be thoroughly investigated. Our current study utilized limited animal numbers, especially since sex differences in response to treatment were present. Furthermore, these studies need to be replicated, including employing other neonatal relevant microbial agents, alternative antibiotics, and potentially other anti-inflammatory agents. In order to be most relevant for both term and preterm human neonates, application of our model to more mature animals that have completed nephrogenesis, e.g. 7 days or older pre-weaning pups, will also be required.

In summary, we developed a novel early-life neonatal murine bacterial sepsis-induced AKI model, and presented initial promising findings that support the use of adjunctive PTX in addition to antibiotics to mitigate AKI. This model may further be suitable to test other treatment modalities targeted against neonatal sepsis-induced AKI.

## Data Availability

The raw data supporting the conclusions of this article will be made available by the authors, without undue reservation.

## Supplementary material

Supplementary Figure 1 GENT and combined (GENT and PTX) inhibit renal tissue cytokine expression in murine neonatal *E. coli* sepsis. Newborn mice less than 24 hours old were intravenously injected with 10^5^ CFUs live *E. coli* K1 strain, followed 1.5 hours later by treatment with gentamicin (GENT, n = 18), pentoxifylline (PTX, n = 23), combined (GENT and PTX [G+P}, n = 24) or an equal volume of sterile saline (SAL, n = 33) (representing untreated sepsis). Renal tissues were harvested 5.5 hours after bacterial injections, and inflammatory cytokines measured in supernatants from homogenized renal tissues, including **(A)** IL-1α, **(B)** IL-12 (p40), **(C)** IL-17, and **(D)** G-CSF. Cytokine concentrations were normalized to pg per mg protein. Mean cytokine concentrations in septic untreated mice were represented through the interrupted line on each panel. Significant concentration differences between treated *vs* untreated septic mice were indicated through triangles above the respective treatment column, whereas significant differences between treatment options were indicated through stars above connecting lines. (*) noticeable but non-significant difference p≥0.05, *p<0.05, ***p<0.001, (Δ) noticeable but non-significant difference p≥0.05, Δ p<0.05, ΔΔ p<0.01, ΔΔΔ p<0.001. **(A)**: p= 0.052 for (Δ) and p=0.059 for (*); **(B)**: p=0.158 for (Δ); and **(C)**: p=0.060 for (Δ) and p= 0.068 for (*).

Supplementary Figure 2 Quantification of fluorescent immunostaining for NGAL from whole renal scans with ImageJ. Immunofluorescent images of formalin-fixed paraffin-embedded kidney sections from 7 days old mice pups 12h after intraperitoneal injection of *E. coli* (10^5^ CFU/g body weight) and anti-NGAL (cat. # ab216462, *Abcam; Waltham, MA*) labeling, Alexa Fluor 568-conjugated secondary antibody, Hoechst (*AnaSpec, Inc.; Fremont, CA*), and fluorescein-conjugated *Lotus Tetragonolobus* lectin (*Vector Laboratories, Inc.; Newark, CA*) staining. **Supplementary Figure S2A** represents an example of a whole renal scan, which was used for quantification of fluorescent staining analysis with ImageJ (red: NGAL, green: *Lotus Tetragonolobus* lectin; blue: DAPI). As illustrated in **Supplementary Figure S2B**, regions of interest (ROIs) were manually placed to outline the renal cortex, excluding any larger cystic structures if present, followed by 3-color image acquisition, and fluorescence intensity and area measurements within ROIs using ImageJ software. As indicated in the representative **Supplementary Figure S2C**, septic mice showed low (arrow 1), medium (arrow 2) and high (arrow 3) intensity NGAL-staining of tubules, whereas control mice demonstrated primarily low intensity NGAL-staining (not shown). Consequently, fluorescence intensity measurements were better able to differentiate NGAL expression between septic and control pups as opposed to areas of fluorescence above threshold.

Supplementary Figure 3 KIM-1 staining of proximal tubules in 7 days old pups. Immunofluorescent images of formalin-fixed paraffin-embedded kidney sections from a 7 days old control mouse **(A)**) as well as *E. coli*-septic pups treated with CEF **(B)**) or combined CEF and PTX **(C)**) 12h after intraperitoneal injection of *E. coli* (10^5^ CFU/g body weight) followed by anti-KIM-1 (cat. # AF1817, *R&D Systems; Minneapolis, MN*) labeling, Alexa Fluor 568-conjugated secondary antibody, Hoechst (*AnaSpec, Inc.; Fremont, CA*), and fluorescein-conjugated *Lotus Tetragonolobus* lectin (*Vector Laboratories, Inc.; Newark, CA*) staining (red: KIM-1; green: *Lotus Tetragonolobus* lectin; blue: DAPI) at 20x magnification. Whereas most controls and septic mice treated with CEF or combined CEF and PTX showed no tubular anti-KIM-1 staining (*see* **Figure 10**), the pups shown in **(A–C)** represented outliers with moderate degrees of anti-KIM-1 staining.
