# Supplementary figures and images for: Attenuation of acute kidney injury in a murine model of neonatal Escherichia coli sepsis
Source: Front Cell Infect Microbiol. 2025 Feb 3;14:1507914. doi: 10.3389/fcimb.2024.1507914 (PMC11830670; doi:10.3389/fcimb.2024.1507914)

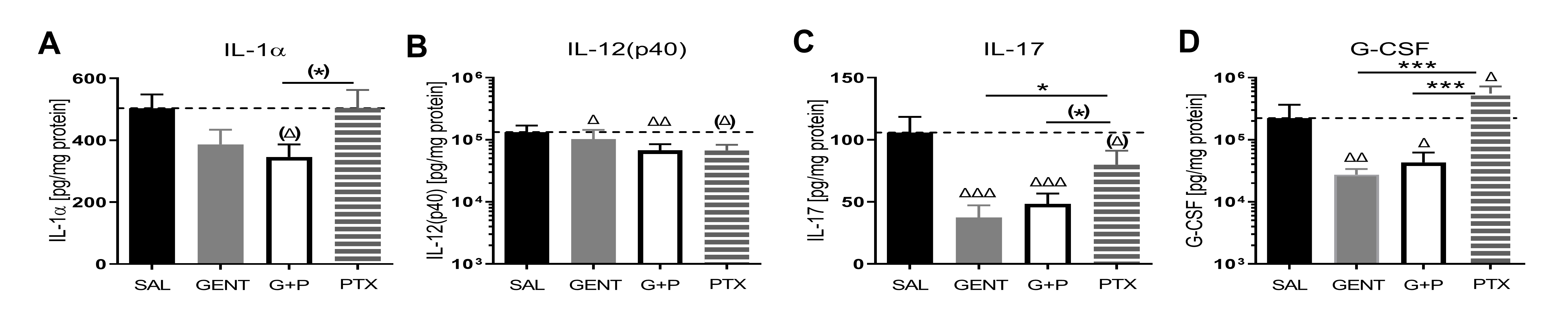

Supplement: Supplementary Figure 1 — GENT and combined (GENT and PTX) inhibit renal tissue cytokine expression in murine neonatal E. coli sepsis. Newborn mice less than 24 hours old were intravenously injected with 105 CFUs live E. coli K1 strain, followed 1.5 hours later by treatment with gentamicin (GENT, n = 18), pentoxifylline (PTX, n = 23), combined (GENT and PTX [G+P}, n = 24) or an equal volume of sterile saline (SAL, n = 33) (representing untreated sepsis). Renal tissues were harvested 5.5 hours after bacterial injections, and inflammatory cytokines measured in supernatants from homogenized renal tissues, including (A) IL-1α, (B) IL-12 (p40), (C) IL-17, and (D) G-CSF. Cytokine concentrations were normalized to pg per mg protein. Mean cytokine concentrations in septic untreated mice were represented through the interrupted line on each panel. Significant concentration differences between treated vs untreated septic mice were indicated through triangles above the respective treatment column, whereas significant differences between treatment options were indicated through stars above connecting lines. (*) noticeable but non-significant difference p≥0.05, *p<0.05, ***p<0.001, (Δ) noticeable but non-significant difference p≥0.05, Δ p<0.05, ΔΔ p<0.01, ΔΔΔ p<0.001. (A): p= 0.052 for (Δ) and p=0.059 for (*); (B): p=0.158 for (Δ); and (C): p=0.060 for (Δ) and p= 0.068 for (*). [file Image1.tif]

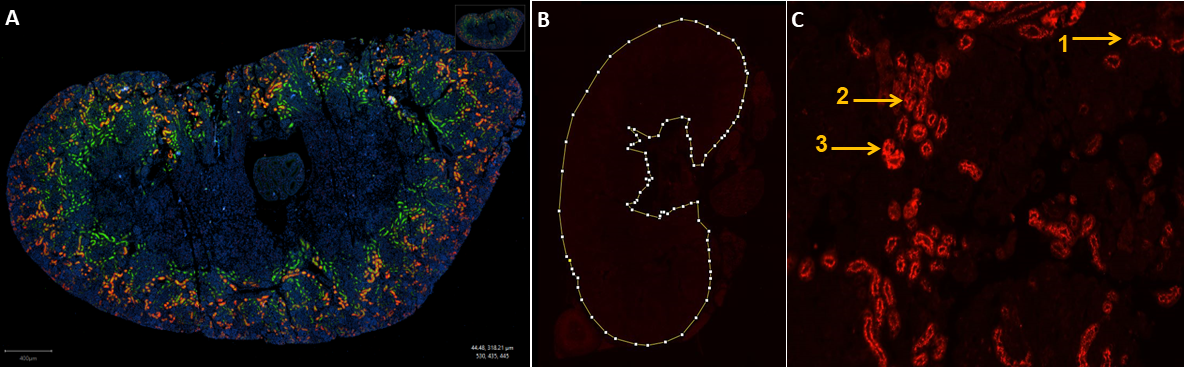

Supplement: Supplementary Figure 2 — Quantification of fluorescent immunostaining for NGAL from whole renal scans with ImageJ. Immunofluorescent images of formalin-fixed paraffin-embedded kidney sections from 7 days old mice pups 12h after intraperitoneal injection of E. coli (105 CFU/g body weight) and anti-NGAL (cat. # ab216462, Abcam; Waltham, MA) labeling, Alexa Fluor 568-conjugated secondary antibody, Hoechst (AnaSpec, Inc.; Fremont, CA), and fluorescein-conjugated Lotus Tetragonolobus lectin (Vector Laboratories, Inc.; Newark, CA) staining. Supplementary Figure S2A represents an example of a whole renal scan, which was used for quantification of fluorescent staining analysis with ImageJ (red: NGAL, green: Lotus Tetragonolobus lectin; blue: DAPI). As illustrated in Supplementary Figure S2B , regions of interest (ROIs) were manually placed to outline the renal cortex, excluding any larger cystic structures if present, followed by 3-color image acquisition, and fluorescence intensity and area measurements within ROIs using ImageJ software. As indicated in the representative Supplementary Figure S2C , septic mice showed low (arrow 1), medium (arrow 2) and high (arrow 3) intensity NGAL-staining of tubules, whereas control mice demonstrated primarily low intensity NGAL-staining (not shown). Consequently, fluorescence intensity measurements were better able to differentiate NGAL expression between septic and control pups as opposed to areas of fluorescence above threshold. [file Image2.tif]

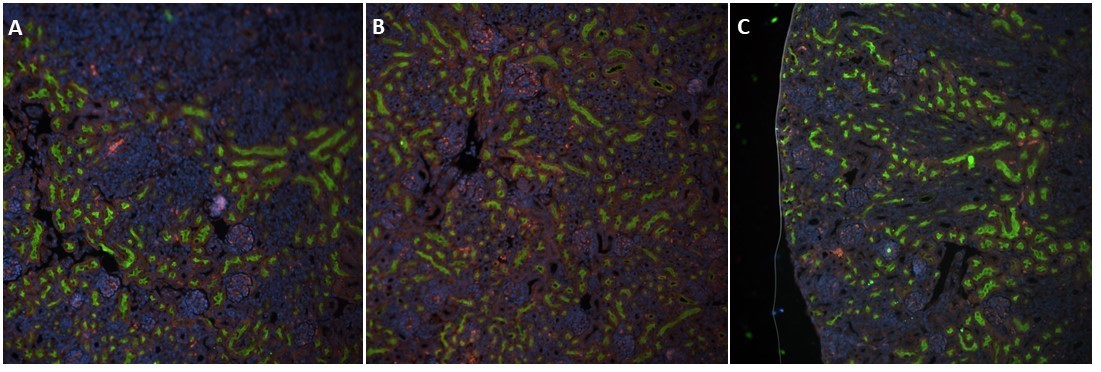

Supplement: Supplementary Figure 3 — KIM-1 staining of proximal tubules in 7 days old pups. Immunofluorescent images of formalin-fixed paraffin-embedded kidney sections from a 7 days old control mouse (A)) as well as E. coli-septic pups treated with CEF (B)) or combined CEF and PTX (C)) 12h after intraperitoneal injection of E. coli (105 CFU/g body weight) followed by anti-KIM-1 (cat. # AF1817, R&D Systems; Minneapolis, MN) labeling, Alexa Fluor 568-conjugated secondary antibody, Hoechst (AnaSpec, Inc.; Fremont, CA), and fluorescein-conjugated Lotus Tetragonolobus lectin (Vector Laboratories, Inc.; Newark, CA) staining (red: KIM-1; green: Lotus Tetragonolobus lectin; blue: DAPI) at 20x magnification. Whereas most controls and septic mice treated with CEF or combined CEF and PTX showed no tubular anti-KIM-1 staining (see Figure 10 ), the pups shown in (A–C) represented outliers with moderate degrees of anti-KIM-1 staining. [file Image3.jpeg]
