# Supplementary material for: Attenuation of acute kidney injury in a murine model of neonatal Escherichia coli sepsis
Source: Front Cell Infect Microbiol. 2025 Feb 3;14:1507914. doi: 10.3389/fcimb.2024.1507914 (PMC11830670; doi:10.3389/fcimb.2024.1507914)
Supplement: Supplementary file 4 [file Table1.docx]

## Supplemental Table 1: Pairwise comparison of renal tissue cytokines between treatment groups within each sex cohort based on linear mixed effect models with adjustment of the interaction term (sex*treatment)

| **Analyte** | **Female** | **Male** |
| --- | --- | --- |
| **Kidney** | **Treatment group** | **Treatment group** |
| IL-1α | **G+P < GENT*** | **G+P > GENT*** |
|  | G+P ≈ PTX | G+P ≈ PTX |
|  | **G+P < SAL*** | G+P ≈ SAL |
|  | GENT ≈ PTX | **GENT < PTX**** |
|  | GENT ≈ SAL | **GENT < SAL**** |
|  | PTX ≈ SAL | PTX ≈ SAL |
| IL-12(p40) | **G+P < GENT**** | **G+P > GENT*** |
|  | G+P ≈ PTX | G+P ≈ PTX |
|  | **G+P < SAL***** | G+P ≈ SAL |
|  | GENT ≈ PTX | **GENT < PTX**** |
|  | GENT ≈ SAL | **GENT < SAL**** |
|  | **PTX < SAL*** | PTX ≈ SAL |
| IL-17 | G+P ≈ GENT | G+P ≈ GENT |
|  | **G+P < PTX*** | G+P ≈ PTX |
|  | **G+P < SAL***** | G+P ≈ SAL |
|  | GENT ≈ PTX | GENT ≈ PTX |
|  | **GENT < SAL**** | **GENT < SAL**** |
|  | PTX ≈ SAL | PTX ≈ SAL |
| G-CSF | G+P ≈ GENT | **G+P > GENT*** |
|  | **G+P < PTX**** | **G+P < PTX**** |
|  | **G+P < SAL**** | G+P ≈ SAL |
|  | **GENT < PTX*** | **GENT < PTX***** |
|  | **GENT < SAL*** | **GENT < SAL**** |
|  | PTX ≈ SAL | **PTX > SAL**** |

> indicates that the first treatment group showed higher cytokine concentrations compared to the second treatment of the respective comparison.

**<** indicates that the first treatment group showed lower cytokine concentrations compared to the second treatment of the respective comparison.

≈ indicates no significant difference between treatment groups.

P-values were derived from t-tests based on linear mixed effect models. Significant comparisons were indicated in **bold**, and significance levels were represented by asterisks: *p<0.05, **p<0.01, ***p<0.001.
